# Effectiveness of Nasal Continuous Positive Airway Pressure vs Nasal Intermittent Positive Pressure Ventilation vs Noninvasive High-Frequency Oscillatory Ventilation as Support After Extubation of Neonates Born Extremely Preterm or With More Severe Respiratory Failure: A Secondary Analysis of a Randomized Clinical Trial

**DOI:** 10.1001/jamanetworkopen.2023.21644

**Published:** 2023-07-03

**Authors:** Xingwang Zhu, Fang Li, Yuan Shi, Zhichun Feng, Daniele De Luca

**Affiliations:** 1 Children’s Hospital of Chongqing Medical University, Ministry of Education Key Laboratory of Child Development and Disorders, Key Laboratory of Pediatrics, Chongqing, China; 2 Bishan Maternal and Child Health Care Hospital, Chongqing, China; 3 Women and Children’s Hospital of Chongqing Medical University, Chongqing, China; 4 Department of Neonatology, Faculty of Pediatrics, the Seventh Medical Center, Chinese PLA General Hospital, Beijing, China; 5 Division of Pediatrics and Neonatal Critical Care, APHP, Paris Saclay University Hospitals, Medical Centre A. Béclère, Paris, France; 6 Physiopathology and Therapeutic Innovation, INSERM U999 Unit, Paris Saclay University, Paris, France

## Abstract

**Importance:**

The NASONE (Nasal Oscillation Post-Extubation) trial showed that noninvasive high-frequency oscillatory ventilation (NHFOV) slightly reduces the duration of invasive mechanical ventilation (IMV) in preterm infants, whereas NHFOV and noninvasive intermittent positive pressure ventilation (NIPPV) result in fewer reintubations than nasal continuous positive airway pressure (NCPAP). It is unknown whether NHFOV is similarly effective in extremely preterm neonates or in those with more severe respiratory failure (based on the duration of previous ventilation and CO_2_ levels).

**Objective:**

To clarify whether NHFOV is better than NIPPV and NCPAP in reducing the duration of IMV in extremely preterm neonates or those with severe respiratory failure.

**Design, Setting, and Participants:**

This study is a predefined secondary analyses of a multicenter randomized clinical trial that was performed at tertiary academic neonatal intensive care units (NICUs) in China. Participants included neonates enrolled in the NASONE trial between December 2017 and May 2021 and belonging to 3 predefined subgroups: (1) born at less than or equal to 28 weeks’ (plus 6 days) gestation, (2) invasively ventilated for more than 1 week from birth, and (3) with CO_2 _greater than 50 mm Hg before or in the 24 hours after extubation. Data analysis was performed in August 2022.

**Intervention:**

NCPAP, NIPPV, or NHFOV since the first extubation and until NICU discharge, with airway pressure higher in NHFOV than in NIPPV and than in NCPAP.

**Main Outcomes and Measures:**

The co–primary outcomes were total duration of IMV during the NICU stay, need for reintubation, and ventilator-free days calculated as per the original trial protocol. Outcomes were analyzed on an intention-to-treat basis as for the whole trial, and subgroup analyses followed the original statistical plan.

**Results:**

Among 1137 preterm infants, 455 (279 boys [61.3%]) were born at 28 weeks’ gestation or less, 375 (218 boys [58.1%]) underwent IMV for more than 1 week from birth, and 307 (183 boys [59.6%]) had CO_2 _greater than 50 mm Hg before or in the 24 hours after extubation. Both NIPPV and NHFOV were associated with significantly fewer reintubations (risk difference range, −28% [95% CI, −39% to −17%] to −15% [95% CI, −25% to −4%]; number needed to treat, 3-7 infants) and early reintubations (risk difference range, −24% [95% CI, −35% to −14%] to −20% [95% CI, −30% to −10%]) than NCPAP, and these reintubations were less frequently due to refractory hypoxemia. IMV was shorter in the NIPPV and NHFOV groups (mean difference range, −5.0 days [95% CI, −6.8 to −3.1 days] to −2.3 days [95% CI, −4.1 to −0.4 days]) than in the NCPAP group. Co–primary outcomes were not different between NIPPV and NHFOV; there was no significant interaction effect. Infants in the NHFOV group showed significantly less moderate-to-severe bronchopulmonary dysplasia than infants in the NCPAP group (range, −12% to −10%; number needed to treat, 8-9 infants) and better postextubation gas exchange in all subgroups. The 3 interventions were provided at different mean airway pressure and were equally safe.

**Conclusions and Relevance:**

The subgroup analyses of extremely preterm or more ill infants confirm the results obtained in the whole population: NIPPV and NHFOV appeared equally effective in reducing duration of IMV compared with NCPAP.

**Trial Registration:**

ClinicalTrials.gov Identifier: NCT03181958

## Key Points

**Question:**

Is nasal continuous positive airway pressure (NCPAP), nasal intermittent positive pressure ventilation (NIPPV), or noninvasive high-frequency oscillatory ventilation (NHFOV) more beneficial in reducing invasive mechanical ventilation (IMV) in neonates?

**Findings:**

This secondary analysis of a randomized clinical trial involving 1137 neonates found that neonates receiving NIPPV or NHFOV had fewer reintubations (range, −28% to −15%), fewer early (within 48 hours after extubation) reintubations (range, −24% to −20%), and shorter duration of IMV (range, −5.0 to −2.3 days) than those supported with NCPAP.

**Meaning:**

These results suggest that NIPPV and NHFOV are essentially similar, and better than NCPAP, in terms of reintubation and duration of IMV in extremely preterm neonates and those with more severe respiratory failure.

## Teaser

This secondary analysis of a randomized clinical trial examines whether noninvasive high-frequency oscillatory ventilation (NHFOV) is better than nasal intermittent positive pressure ventilation (NIPPV) and nasal continuous positive airway pressure (NCPAP) in reducing the duration of invasive mechanical ventilation in extremely preterm infants and those with severe respiratory failure.

## Introduction

Invasive mechanical ventilation (IMV) is a life-saving intervention for preterm neonates but is associated with negative outcomes, such as bronchopulmonary dysplasia (BPD) and later neurologic impairment.^1,2^ Several techniques of noninvasive respiratory support have been proposed to decrease the use of IMV in preterm neonates, but it is still unclear which techniques should be preferred and integrated into clinical protocols. In fact, it is likely that 1 solution does not fit all and that respiratory support needs to be tailored to gestational age and clinical severity.

The NASONE (Nasal Oscillation Post-Extubation) study was a multicenter, assessor-blinded, randomized clinical trial investigating, from primary extubation until discharge from the neonatal intensive care unit (NICU), the use of noninvasive high-frequency oscillatory ventilation (NHFOV) vs nasal continuous positive airway pressure (NCPAP) vs unsynchronized noninvasive intermittent positive pressure ventilation (NIPPV) to reduce the duration of IMV.^3^ One of the biggest neonatal ventilation trials ever conducted (1440 enrolled patients), NASONE received independent public funding^4^ and used as much as possible the rigorous methods of pharmacological trials, as well as field training on the interventions.^3^ The trial was also pathophysiology based, because NHFOV and NIPPV management was guided by respiratory physiology and mechanics^5,6,7,8,9,10^ to generate higher mean airway pressure than in the NCPAP group. The NASONE study found that NHFOV slightly reduces the duration of IMV, whereas both NHFOV and NIPPV provide a lower risk of reintubation compared with NCPAP, and that the 3 respiratory support techniques were equally safe.^11^ These results were obtained in patients with gestational age less than or equal to 32 weeks, but the NASONE protocol^3^ anticipated some predefined subgroup analyses for certain categories of patients. These were the extremely preterm (≤28 weeks’ gestation) neonates and those with more severe respiratory failure. The latter group was defined as those undergoing IMV for at least 1 week continuously from birth or with CO_2 _greater than 50 mm Hg before or in the 24 hours after extubation. Patients with these characteristics are more likely to require long IMV.^12,13^ These subgroups were chosen by consensus between the investigators to identify patients who were more likely to undergo IMV.^14^

Clinical algorithms integrating respiratory support have been proposed to personalize the assistance of extremely preterm neonates, but we need evidence-based data to refine them.^15^ We report the results of the predefined subgroup analyses of the NASONE trial: we hypothesize that NHFOV, when used from primary extubation until NICU discharge, is more efficacious than NCPAP or NIPPV in reducing the need for IMV in extremely preterm neonates or in those with more severe respiratory failure.

## Methods

### Study Design

The trial protocol was previously published,^3^ and all methodological details are available there and in Supplement 1. The trial was granted ethical approval by the ethics committee of the Third Affiliated Hospital of Chongqing Military Medical University, and informed consent was obtained from parents or guardians antenatally or upon NICU admission. This report was prepared following Consolidated Standards of Reporting Trials (CONSORT) reporting guidelines for multigroup trials.^16^

Sixty-nine third-level referral NICUs in China participated. Between December 2017 and May 2021, using a software-generated random number sequence posted on a dedicated website, neonates were quickly randomized to receive NCPAP, NIPPV, or NHFOV, when extubation was imminent (within 1 hour). Randomization was not performed earlier to avoid any bias due to knowledge of the assigned intervention. Patients were allocated to a study group immediately after extubation and until NICU discharge (ie, no crossover was allowed; in the case of reintubation, when the neonate was re-extubated, the same treatment was provided). Devices and interfaces used to provide the respiratory support have been described elsewhere.^3,11^ The trial was originally powered to detect a 20% reduction in IMV duration for NHFOV-treated patients.^3^

### Subgroup Analysis and Outcomes

According to the predefined criteria,^3^ we subdivided the NASONE population into 3 groups of neonates: (1) those born at a gestational age 28 weeks plus 6 days (28^+6^) or earlier or after 28^+6^ weeks, (2) those invasively ventilated for less than 1 week or 1 week or longer continuously from birth, and (3) those with CO_2 _less than or equal to or greater than 50 mm Hg before or in the 24 hours after extubation (CO_2_ was measured using arterialized capillary blood gas analysis and/or transcutaneous monitoring following the American Association for Respiratory Care guidelines^17^ and the manufacturer’s recommendations). The subgroups of neonates of gestational age 28 weeks or less or invasively ventilated for 1 week or longer continuously from birth or having CO_2 _greater than 50 mm Hg were considered as subgroups of interest.

For the subgroups of interest, we analyzed and reported the same co–primary outcomes chosen for the whole trial: (1) total duration of IMV during the NICU stay, (2) need for reintubation (criteria for reintubation were fixed and described in the protocol^3^), and (3) ventilator-free days (VFD) calculated as per the trial protocol.^3^ We also studied the need for early (ie, within 48 hours from extubation) reintubation, because this is considered likely to be directly linked to the original respiratory disorder.^18,19^ These outcomes address the global burden of care due to IMV, because they describe it from different clinical standpoints.

For the whole population, we also analyzed secondary efficacy (ie, prevalence of BPD and moderate-to-severe BPD, use of postnatal steroids, in-hospital mortality, and a BPD-mortality composite end point) and safety, including prevalence of air leaks and apneic events, patient comfort, weekly weight gain, prevalence of severe nasal skin injury, grade III to IV intraventricular hemorrhage, stage II to III necrotizing enterocolitis, and retinopathy of prematurity, if retinopathy of prematurity grade was more than 2 outcomes. The definitions and monitoring strategies for these outcomes were previously described.^3^ Patient comfort was evaluated with the Premature Infant Pain Profile score^20^ calculated in the first 48 hours from the onset of study intervention, and nasal injury was evaluated with a dedicated visual score.^21^ Finally, we analyzed the duration of the study intervention and supplemental oxygen.

### Statistical Analysis

Data analysis was performed in August 2022. Basic patient characteristics were compared between the study groups, within each subgroup of interest, using χ^2^ test and 1-way analysis of variance followed by the Sidak post hoc test, adjusting for multiple comparisons (when the overall analysis of variance results were significant). Outcomes were analyzed on an intention-to-treat basis as for the whole trial and subgroup analyses followed the original statistical analysis plan.^3,11^ In detail, for dichotomous outcomes, we calculated a risk difference (95% CI), whereas for continuous outcomes, we calculated the mean difference (95% CI) between the groups. The need for reintubation was also studied with Kaplan-Meier analysis contrasted with the log-rank test. Interaction analyses for the co–primary outcomes were performed with Cox or linear regressions for dichotomous (reintubations) or continuous variables (duration of IMV or VFD), respectively; an interaction term between the study intervention and the variable used to define subgroups was inserted per each regression model, and results were reported as hazard ratio (95% CI) or β (95% CI) for dichotomous or continuous variables, respectively.^22^ The number needed to treat (NNT) was also calculated, where appropriate. Following the European Medicines Agency guidelines on the investigation of subgroups in clinical trials,^23^ data from all subgroups were presented together and visually analyzed using multiple forest plots, which were obtained using Open Meta-Analyst 10.1.^24^ Statistical analyses were performed with SPSS statistical software version 16 (IBM) and GPower software version 3.1.9.6 (University of Dusseldorf). Two-sided *P* < .05 was considered statistically significant.

## Results

A total of 1137 preterm infants were included in the study: 455 (279 boys [61.3%]) were born at 28 weeks’ gestation or less, 375 (218 boys [58.1%]) underwent IMV for 1 week or longer from birth, and 307 (183 boys [59.6%]) had CO_2 _greater than 50 mm Hg before or in the 24 hours after extubation (eFigure 1 in Supplement 2). The trial groups were balanced in terms of basic characteristics in the subgroups of interest except for birth weight (Table). Basic characteristics for the alternate subgroups are shown in eTable 1 in Supplement 2. The maximal applied airway pressure was 8 cm H_2_O in the NCPAP group, 14 cm H_2_O in the NIPPV group, and 16 cm H_2_O in the NHFOV group.

**Table.** Characteristics of Neonates in the 3 Subgroups of Interest

Characteristic	Neonates, No. (%) (N = 1337)											
	≤28 wk Gestation (n = 455)				Ventilated ≥1 wk from birth (n = 375)				CO_2 _>50 mm Hg before or in the 24 h after extubation (n = 307)			
	NCPAP (n = 134)	NIPPV (n = 160)	NHFOV (n = 161)	*P* value	NCPAP (n = 110)	NIPPV (n = 131)	NHFOV (n = 134)	*P* value	NCPAP (n = 123)	NIPPV (n = 94)	NHFOV (n = 90)	*P* value
Gestational age, mean (SD), wk	27.3 (0.9)	27.3 (0.9)	27.2 (0.9)	.76	28.7 (1.8)	28.9 (1.9)	28.8 (1.8)	.82	29.1 (1.9)	29 (1.9)	28.6 (1.8)	.20
Birth weight, mean (SD), g	1085 (202)	1040 (194)	1033 (186)	.05	1205 (325)	1271 (393)	1183 (291)	.09	1234 (293)	1309 (395)	1252 (323)	.04
Sex												
Male	81 (60.4)	100 (62.5)	98 (60.8)	.93	58 (52.7)	79 (60.3)	81 (60.4)	.39	69 (56.1)	59 (62.7)	55 (61.1)	.57
Female	53 (39.6)	60 (37.5)	63 (39.1)		52 (47.3)	52 (39.7)	53 (39.6)		54 (43.9)	35 (37.2)	35 (38.9)	
Small for gestational age	9 (6.7)	13 (8.1)	15 (9.3)	.72	10 (9.1)	8 (6.1)	14 (10.4)	.43	11 (8.9)	6 (6.4)	10 (11.1)	.52
Twins	38 (28.3)	48 (30)	51 (31.7)	.82	34 (30.9)	35 (26.7)	45 (33.6)	.47	30 (24.4)	19 (20.2)	21 (23.3)	.76
Cesarean delivery	49 (36.5)	58 (36.2)	56 (34.7)	.94	57 (51.8)	72 (54.9)	80 (59.7)	.45	68 (55.3)	45 (47.9)	52 (57.8)	.36
Prenatal steroids^a^	63 (47.0)	74 (46.2)	81 (50.3)	.74	46 (41.8)	54 (41.2)	49 (36.5)	.64	58 (47.1)	41 (45)	39 (43.3)	.82
Clinical Risk Index for Babies-II score, mean (SD)	7.8 (3.1)	7.8 (3.2)	8.2 (2.8)	.31	6.7 (3.3)	6.2 (3.7)	6.5 (3.1)	.47	5.9 (3.2)	5.9 (3.6)	6.1 (3.4)	.88
5-Factor Apgar score, median (IQR)	8 (7-9)	8 (7-9)	9 (8-9)	.31	9 (8-9)	8 (7-9)	9 (8-9)	.34	9 (8-9)	8 (7-10)	9 (8-9)	.20
Surfactant replacement^b^	121 (90.3)	151 (94.4)	145 (90.1)	.30	99 (90)	121 (92.4)	121 (90.3)	.77	109 (88.6)	87 (92.5)	82 (91.1)	.60
Early-onset sepsis	3 (2.2)	7 (4.4)	3 (1.9)	.35	5 (4.5)	5 (3.8)	2 (1.5)	.35	6 (4.9)	6 (6.4)	4 (4.4)	.82
Postnatal age at extubation, median (IQR), d	4 (2-8)	4 (2-8)	5 (2-8)	.13	10 (8-15)	10 (8-13)	9 (7-12)	.51	5 (3-9)	6 (3-9)	5 (3-8)	.23
Oxygenation index at extubation, mean (SD)^c^^,^^d^	3.7 (1.8)	3.7 (2.5)	4.0 (2.8)	.53	4.2 (2.4)	4.1 (2.5)	4.1 (2.7)	.96	3.7 (2.2)	4.5 (3.0)	4.7 (3.8)	.04
pH at extubation, mean (SD)^d^	7.35 (0.11)	7.35 (0.09)	7.34 (0.09)	.24	7.36 (0.11)	7.36 (0.09)	7.35 (0.09)	.92	7.32 (0.10)	7.33 (0.10)	7.29 (0.10)	.07
Partial pressure of carbon dioxide at extubation, mean (SD), mm Hg^d^	40.2 (11.3)	39.2 (10.4)	41.3 (11.3)	.63	41.9 (11.5)	41.3 (11.9)	41.7 (12.2)	.34	46.1 (12.5)	47.5 (13.6)	50.4 (13.1)	.06
Airway pressure at extubation, mean (SD), cm H_2_O	6.7 (0.8)	7.5 (2.1)	7.9 (2.2)	<.001	6.4 (1.6)	8.1 (2.4)	8 (2.0)	<.001	6.6 (2.2)	7.8 (2.2)	8.1 (2)	<.001
Bronchopulmonary dysplasia	99 (73.8)	104 (65)	97 (60.2)	.04	62 (56.4)	70 (53.4)	61 (45.5)	.20	60 (48.8)	43 (45.7)	42 (46.7)	.90

Abbreviations: NCPAP, nasal continuous positive airway pressure; NHFOV, noninvasive high-frequency oscillation ventilation; NIPPV, noninvasive positive pressure ventilation.

^a^ Prenatal steroid is considered to have occurred if dosing was complete (ie, 2 12-mg doses of betamethasone, 24 hours apart from each other).

^b^ Surfactant replacement was always performed by the intubation-surfactant-extubation technique.

^c^ Oxygenation index is calculated as follows: (inspired oxygen faction × mean airway pressure × 100) / partial oxygen pressure.

^d^ Oxygenation index, pH, partial pressure of carbon dioxide, and airway pressure values are those measured at 24 hours from the first extubation, that is from the onset of the trial intervention (ie, the time point for gas analysis and data capture).

**Figure 1.**
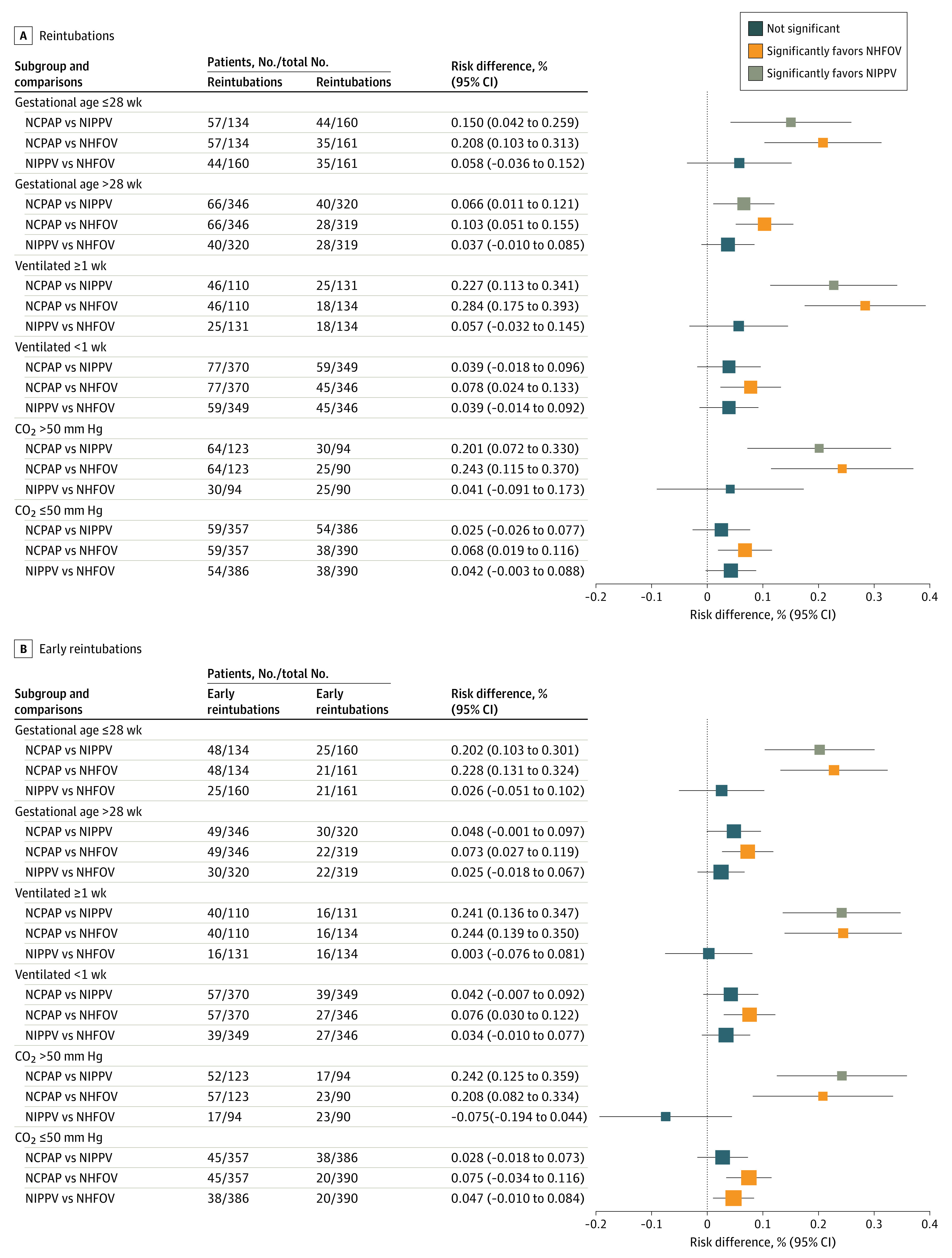
Reintubations and Early (Within 48 Hours From Extubation) Reintubations Squares and lines indicate the percentage risk differences and their 95% CIs. Square size is proportional to the subgroup size. *P* values for all comparisons are shown in the eAppendix in Supplement 2. Results for the whole population have been published elsewhere.^11^

**Figure 2.**
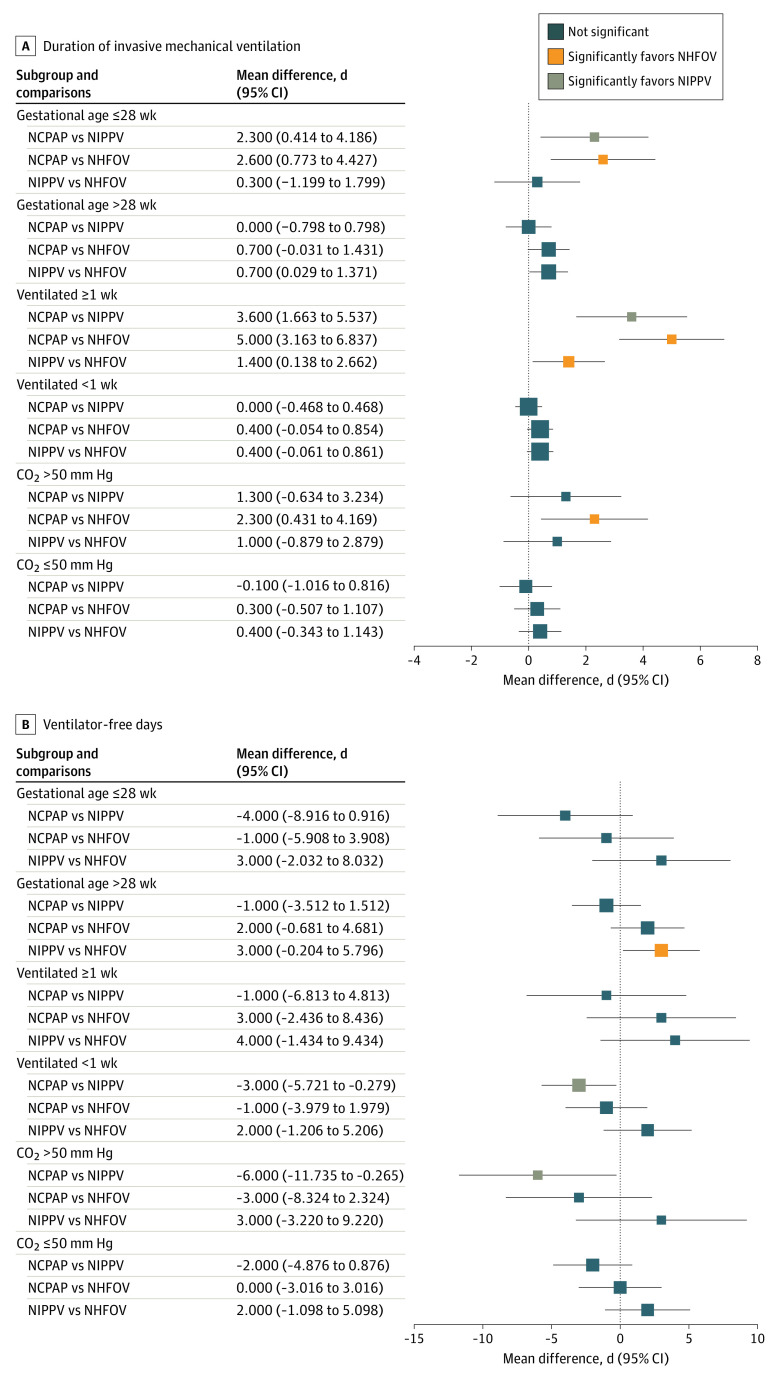
Duration of Invasive Mechanical Ventilation (IMV) and Ventilator-Free Days Both outcomes are measured in days. Data are shown as mean difference and 95% CI and illustrated as forest plots per subgroup. Square size is proportional to the subgroup size. *P* values for all comparisons are shown in the eAppendix in Supplement 2. Results for the whole population have been published elsewhere.^11^ NCPAP indicates nasal continuous positive airway pressure; NHFOV, noninvasive high-frequency oscillation ventilation; and NIPPV, noninvasive positive pressure ventilation.

Figure 1 and Figure 2 show the results for co–primary outcomes (*P* values for comparisons are shown in the eAppendix in Supplement 2). Both NIPPV and NHFOV had fewer reintubations (range, −28% [95% CI, −39% to −17%] to −15% [95% CI, −25% to −4%]) and early reintubations (range, −24% [95% CI, −35% to −14%] to −20% [95% CI, −30% to −10%]) than the NCPAP group in all subgroups of interest with a NNT of 3 to 7 infants (Figure 1); no significant difference was seen between NIPPV and NHFOV. Moreover, reintubations in the NIPPV and NHFOV groups were less frequently attributed to refractory hypoxemia than in the NCPAP group in all subgroups (eTable 2 in Supplement 2). The results were confirmed by Kaplan-Meier analysis, whose curves were significantly different (eFigure 2 in Supplement 2). Similar results were evident in terms of IMV duration, but, in addition, the NHFOV group had a shorter IMV duration than the NIPPV group among neonates invasively ventilated for at least 1 week (Figure 2A). IMV was shorter in the NIPPV and NHFOV groups (mean difference range, −2.3 days [95% CI, −4.1 to −0.4 days] to −5.0 days [95% CI, −6.8 to −3.1 days]) than in the NCPAP group. No consistent differences were seen in subgroups of interest for VFD (Figure 2B). There was no significant interaction effect—that is, the treatment effect on the co–primary outcomes was not influenced by variables used to define the subgroups (eTable 3 in Supplement 2), with the exception of VFD and duration of IMV in extremely preterm and long-term ventilated infants, respectively. These exceptions are, however, unlikely to be clinically meaningful. The expected false-positive rates for co–primary outcomes are reported in eTable 4 in Supplement 2.

**Figure 3.**
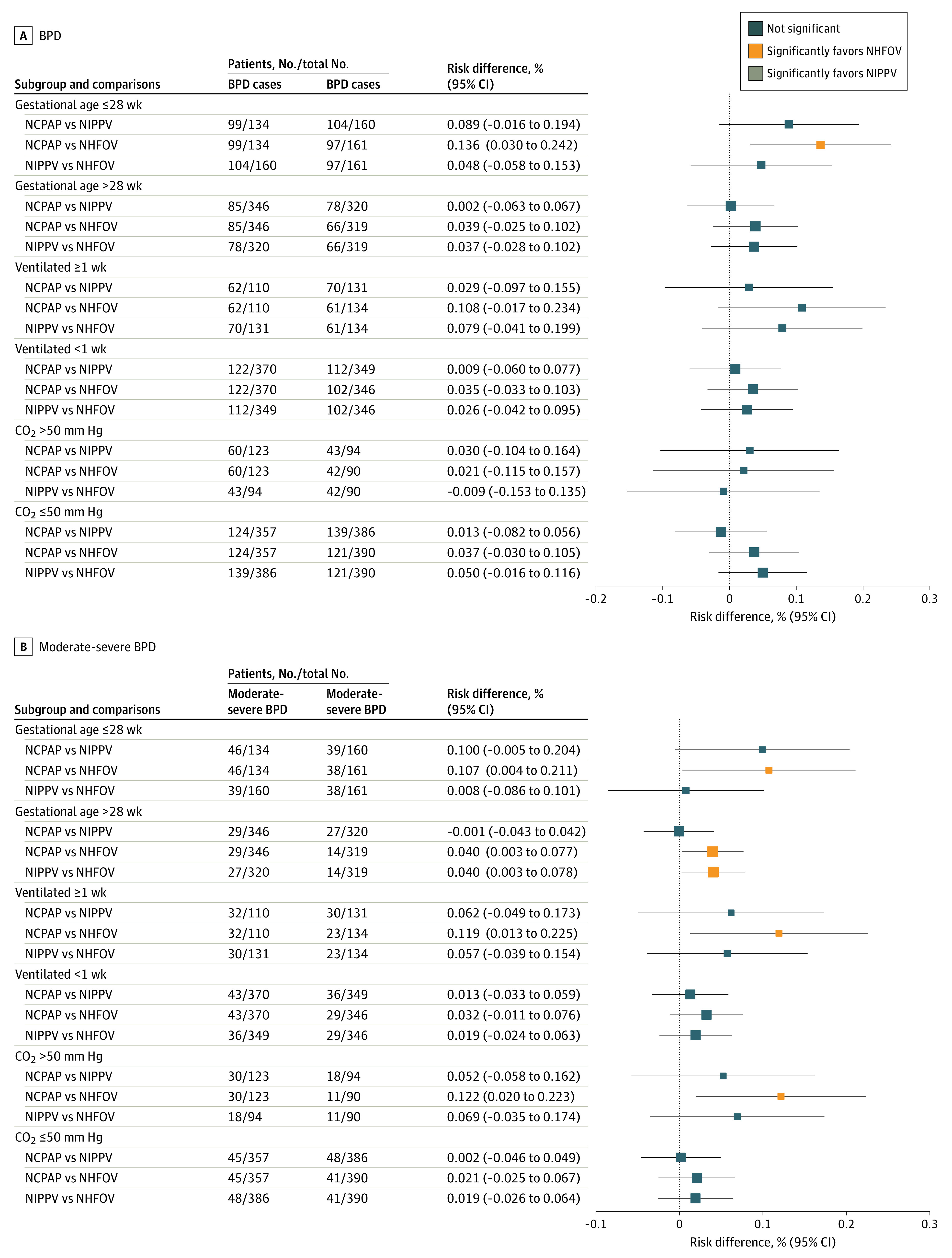
Bronchopulmonary Dysplasia (BPD) and Moderate-to-Severe BPD Squares and lines indicate the mean percentage risk differences and their 95% CI, respectively. Square size is proportional to the subgroup size. *P* values for all comparisons are shown in the eAppendix in Supplement 2. Results for the whole population have been published elsewhere.^11^

Figure 3 shows BPD as secondary outcome: the NHFOV group had less BPD than the NCPAP group in neonates of gestational age 28 weeks or less (Figure 3A). In addition, NHFOV showed less moderate-to-severe BPD than the NCPAP group in all subgroups of interest (range, −12% to −10%; NNT, 8-9 infants) (Figure 3B) (*P* values for comparisons are shown in the eAppendix in Supplement 2).

**Figure 4.**
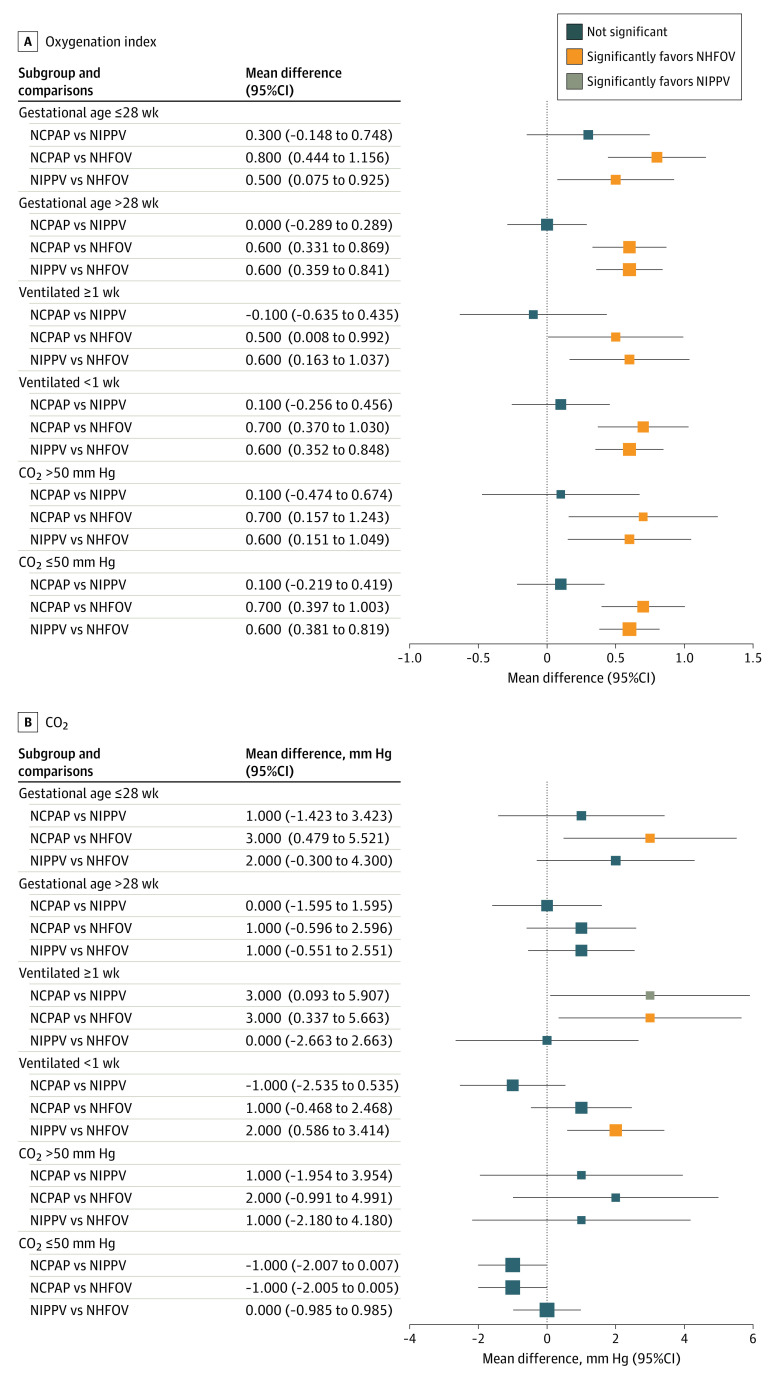
Oxygenation Index and CO_2_ at 24 Hours From the Study Intervention Oxygenation index is a dimensionless variable and CO_2_ is expressed in millimeters of mercury (details on their measurements are available in the trial protocol^3^). Squares and lines indicate the mean differences and their 95% CIs. Square size is proportional to the subgroup size. *P* values for all comparisons are shown in the eAppendix in Supplement 2. Results for the whole population have been published elsewhere.^11^ NCPAP indicates nasal continuous positive airway pressure; NHFOV, noninvasive high-frequency oscillation ventilation; and NIPPV, noninvasive positive pressure ventilation.

Figure 4 shows gas exchange measures at 24 hours from the study intervention (ie, at 24 hours from the first extubation). Infants in the NHFOV group had a better oxygenation index than did infants in the NCPAP or NIPPV groups in all subgroups of interest (Figure 4A); they also showed lower CO_2_ compared with the NCPAP group in neonates of gestational age 28 weeks or less and in those invasively ventilated for at least 1 week. Similar results were observed for NIPPV in neonates ventilated for at least 1 week (Figure 4B). The absolute values of these differences were, however, small (*P* values for comparisons are shown in the eAppendix in Supplement 2).

Findings for the other secondary outcomes are shown in eFigures 3 to 7 in Supplement 2. The NHFOV group showed reduced prevalence of the BPD-mortality composite outcome compared with the NCPAP group in neonates of gestational age 28 weeks or less (eFigure 3C in Supplement 2); no consistent differences were seen for postnatal steroids or in-hospital mortality (eFigures 3A and 3B in Supplement 2). Both NIPPV and NHFOV had fewer apneic events than the CPAP group in neonates invasively ventilated for at least 1 week and in those with CO_2 _greater than 50 mm Hg before or in the 24 hours after extubation (eFigure 4B in Supplement 2). No consistent differences were seen regarding air leaks and patient comfort (eFigures 5A-5C in Supplement 2), weekly weight gain and nasal skin injury (eFigures 5A and 5B in Supplement 2), prematurity-related extrapulmonary complications (eFigures 6A-6C in Supplement 2), and duration of oxygen supplementation and study intervention (eFigures 7A and 7B in Supplement 2).

## Discussion

This secondary analysis of a randomized clinical trial was focused on extremely preterm neonates and those with more severe respiratory failure given their higher risk of negative outcomes. We found results similar to those reported in the whole population.^11^ In detail, we found that (1) both NIPPV and NHFOV were associated with fewer reintubations, fewer early reintubations, and shorter IMV duration compared with NCPAP in all subgroups of interest; (2) NHFOV was associated with less moderate-to-severe BPD than NCPAP in all subgroups of interest; (3) patients treated with NHFOV showed significantly better postextubation gas exchange than those treated with NCPAP or NIPPV, consistent with data showing that reintubations were less frequently due to refractory hypoxemia in the NHFOV group (the absolute gas exchange improvement was, however, small); and (4) NCPAP, NIPPV, and NHFOV were equally safe. These results confirm the main NASONE trial results and help tailoring the respiratory support strategy to extremely preterm or severely ill infants.

The hypothesis that, in these subgroups, NHFOV would be more efficacious than the other techniques to reduce the duration of IMV is disproven. Because there are several respiratory techniques available, it is clear that there is no single solution for all patients.^15^ Conversely, we can consider that NIPPV and NHFOV are essentially similar, and better than NCPAP, in terms of reintubation and total duration of IMV during the whole NICU stay. These results are similar to those obtained in the whole population,^11^ as essentially there is no interaction effect. The effect size in the subgroups of interest seems slightly bigger than that of the whole population.^11^ In fact, extremely preterm or more ill infants treated with NIPPV or NHFOV have a reintubation risk reduced by 15% to 28% and an IMV duration shortened by 2.3 to 5.0 days (in the whole population, these were 12% and 1.0-1.5 days, respectively^11^). We used 3 co–primary outcomes to describe the need for reintubation and IMV from various points of view and be as comprehensive as possible. Our findings are consistent with previous trials^25,26^ reporting fewer reintubations with NHFOV or NIPPV. The effect of NIPPV and NHFOV may be clinically relevant because every additional day of IMV^27^ and each reintubation^28^ have been associated with various negative outcomes in similar populations; of note, 2 days of IMV has been associated with significantly increased neutrophil influx into the alveoli.^29^

NHFOV seems to be associated with reduced prevalence of moderate-to-severe BPD and better postextubation gas exchange in all subgroups of interest: this might be explained with the physiology-based ventilatory approach of the NASONE trial. In fact, NHFOV was used with airway pressure higher than in the other groups and using lung recruitment maneuvers, as is usually done during invasive HFOV, without any crossover. Previous trials^30^ have compared ventilatory techniques at equal airway pressure and, unsurprisingly, have not reported any advantage because this prevents full exploitation of the potential of NIPPV and NHFOV. Several findings suggest that a ventilatory strategy with high airway pressure may be crucial to the improvement of respiratory outcomes^11^ and that increasing airway pressure reduces extubation failure also during NCPAP.^31^ Therefore, the higher airway pressure and alveolar recruitment maneuvers allowed in the NHFOV group, rather than the ventilatory mode itself, might be responsible for the results. The high airway pressure may have reduced the proinflammatory trigger represented by IMV.^32^ Furthermore, decreasing reintubations may reduce the occurrence of ventilator-associated pneumonia, which can also increase the risk of BPD.^33^ It was simple to allow higher airway pressure in NHFOV, although this might also be provided with a conventional technique, such as NIPPV. This, however, would require synchronization, which is unfortunately unavailable in most neonatal ventilators. Interestingly, the early use of invasive HFOV was also associated with reduced prevalence of BPD, although the evidence was weakened by inconsistency across trials and the variable case mix of the studied populations.^34^ Several factors, such as different ventilatory strategies and individual patient characteristics, may have interacted and influenced the efficacy of HFOV on a population level. The NASONE subgroup analysis overcomes, at least partially, these factors because it focuses on homogeneous subgroups of patients and applies NHFOV with a well-defined, physiology-based strategy, without any crossover.^3^ Moreover, subgroups were selected according to simple clinical criteria, which facilitates the patient bedside identification.

We also observed a lower incidence of apneas during NIPPV and NHFOV, consistent with what has been suggested in smaller or shorter studies.^15,35,36,37^ Because the respiratory support technique was applied until NICU discharge, these data indicate the efficacy of NIPPV and NHFOV in reducing apneic events overall, not only for a short period following extubation. Nonetheless, the absolute effect size is small, and it is unclear whether this finding can be clinically meaningful. Our findings also showed the equal safety of the 3 trialed techniques in all subgroups.^11^ A higher risk of air leaks, nasal trauma, or discomfort has been excluded, and this is also consistent with data from smaller and shorter NHFOV trials and a recent cross-sectional study showing that NHFOV provides good patient comfort, which was not negatively influenced by the increasing airway pressure.^15,38^ Because crossover was not allowed, we cannot clarify whether patients started on NCPAP would still respond to alternate techniques when NCPAP fails. Nonetheless, the study demonstrates the safety of the trialed techniques; thus, treating with NIPPV or NHFOV an infant for whom NCPAP would not have failed may not entail any relevant harm.

### Limitations

Limitations of this study include those related to the original design of the NASONE trial.^11^ Furthermore, although it was performed with a strict method, this work consisted of many subgroup analyses about 3 study interventions, and sample size calculation was originally performed for one of the co–primary outcomes. Some results may be due to chance as analyses may have reduced power, particularly for the comparisons involving the smaller subgroups: thus, further dedicated trials may be needed to generalize these findings. A center effect has been minimized, but not totally excluded. The definitions of subgroups can be criticized because they were pragmatically chosen to facilitate the recruitment and did not use any complex metrics or tools to characterize patient pathophysiology. The results, notably those regarding moderate-to-severe BPD (which was only a secondary outcome), should be confirmed in specifically designed trials. Subgroups of interest were slightly imbalanced for birth weight, but the NHFOV group had the lowest mean weight; thus, positive results obtained in this group were observed despite this imbalance. Although we comprehensively described the need for IMV with several end points, the trialed intervention might only indirectly affect the total IMV duration because it may be influenced by concurrent factors, including extrapulmonary prematurity-related complications. However, these complications were similar between the groups, and we reduced this influence by using fixed extubation and reintubation criteria, as well as by avoiding crossover.^3^ The improvement in gas exchange and apneas observed with NHFOV is of uncertain clinical value given the small absolute effect, although it is consistent with findings observed in co–primary outcomes. Finally, the exposition to high airway pressure level may deserve further studies to ascertain its long-term safety. The NASONE population consisted of exclusively Chinese infants born at 25 weeks’ or later gestation who did not often receive steroid prophylaxis, remained on IMV for an average of 4 days from birth, and had a relatively high BPD incidence, irrespective of the study interventions. These peculiarities may have influenced our results, particularly for BPD, which may change if its a priori risk is lower.^39^ Thus, the results cannot be directly generalized to populations with different characteristics or to NICUs with different expertise. However, in the NASONE population, the use of IMV and the BPD rate are also quite similar to those of other recent trials, demonstrating that there is a real difficulty in improving this clinical problem.

## Conclusions

In this study, neonates born extremely preterm or with more severe respiratory failure had fewer reintubations, fewer early reintubations, and shorter IMV duration if they received NIPPV or NHFOV, rather than NCPAP, from extubation until NICU discharge. NHFOV may also be associated with less moderate-to-severe BPD than NCPAP. NIPPV and NHFOV are as safe as NCPAP. These findings are similar to those of the whole NASONE population and help tailor respiratory support during NICU stay.

## Notes

Supplement 1.
Trial Protocol

Supplement 2. **eFigure 1.** CONSORT Flowchart of the Original NASONE Trial **eTable 1.** Basic Characteristics of Population Subgroups Complementary to the Subgroups of Interest (i.e.: Subgroups With More Mature Neonates and Those With Less Severe Respiratory Failure) **eAppendix.** *P* Values for Figures **eTable 2.** Reasons for Reintubation in the Subgroups **eTable 3.** Interaction Analyses for All Coprimary Outcomes **eTable 4.** Expected False-Positive Rates for All Coprimary Outcomes **eFigure 2.** Kaplan-Meier Analysis for Reintubations **eFigure 3.** Secondary Outcomes: Use of Postnatal Steroids (A), In-Hospital Mortality (B) and BPD/Mortality (C) **eFigure 4.** Secondary Outcomes: Airleaks (A), Number of Apneas/Week (B) and PIPP Score (C) **eFigure 5.** Secondary Outcomes: Weekly Weight Gain (A) and Severe Nasal Skin Injury (B) **eFigure 6.** Secondary Outcomes: IVH (A), NEC (B) and ROP (C) **eFigure 7.** Secondary Outcomes: Duration of Oxygen Supplementation (A) and of Study Intervention (B)

Supplement 3.
Nonauthor Collaborators

Supplement 4.
Data Sharing Statement

## References

[1] Jensen EA, DeMauro SB, Kornhauser M, Aghai ZH, Greenspan JS, Dysart KC. Effects of multiple ventilation courses and duration of mechanical ventilation on respiratory outcomes in extremely low-birth-weight infants. JAMA Pediatr. 2015;169(11):1011-1017. doi:10.1001/jamapediatrics.2015.2401 26414549 PMC6445387

[2] Vliegenthart RJS, Onland W, van Wassenaer-Leemhuis AG, De Jaegere APM, Aarnoudse-Moens CSH, van Kaam AH. Restricted ventilation associated with reduced neurodevelopmental impairment in preterm infants. Neonatology. 2017;112(2):172-179. doi:10.1159/000471841 28601870 PMC5637296

[3] Shi Y, De Luca D; NASal OscillatioN post-Extubation (NASONE) Study Group. Continuous positive airway pressure (CPAP) vs noninvasive positive pressure ventilation (NIPPV) vs noninvasive high frequency oscillation ventilation (NHFOV) as post-extubation support in preterm neonates: protocol for an assessor-blinded, multicenter, randomized controlled trial. BMC Pediatr. 2019;19(1):256. doi:10.1186/s12887-019-1625-1 31349833 PMC6659219

[4] Shankar-Aguilera S, Taveira M, De Luca D. Neonatal ventilation trials need specific funding. Lancet Respir Med. 2014;2(11):867-869. doi:10.1016/S2213-2600(14)70194-8 25439565

[5] De Luca D, Carnielli VP, Conti G, Piastra M. Noninvasive high frequency oscillatory ventilation through nasal prongs: bench evaluation of efficacy and mechanics. Intensive Care Med. 2010;36(12):2094-2100. doi:10.1007/s00134-010-2054-7 20857278

[6] De Luca D, Piastra M, Pietrini D, Conti G. Effect of amplitude and inspiratory time in a bench model of non-invasive HFOV through nasal prongs. Pediatr Pulmonol. 2012;47(10):1012-1018. doi:10.1002/ppul.22511 22328295

[7] De Luca D, Costa R, Visconti F, Piastra M, Conti G. Oscillation transmission and volume delivery during face mask-delivered HFOV in infants: bench and in vivo study. Pediatr Pulmonol. 2016;51(7):705-712. doi:10.1002/ppul.23403 26918535

[8] De Luca D. Noninvasive high-frequency ventilation and the errors from the past: designing simple trials neglecting complex respiratory physiology. J Perinatol. 2017;37(9):1065-1066. doi:10.1038/jp.2017.84 28904405

[9] Centorrino R, Dell’Orto V, Gitto E, Conti G, De Luca D. Mechanics of nasal mask-delivered HFOV in neonates: a physiologic study. Pediatr Pulmonol. 2019;54(8):1304-1310. doi:10.1002/ppul.24358 31091025

[10] Mukerji A, Finelli M, Belik J. Nasal high-frequency oscillation for lung carbon dioxide clearance in the newborn. Neonatology. 2013;103(3):161-165. doi:10.1159/000345613 23258368

[11] Zhu X, Qi H, Feng Z, Shi Y, De Luca D; Nasal Oscillation Post-Extubation (NASONE) Study Group. Noninvasive high-frequency oscillatory ventilation vs nasal continuous positive airway pressure vs nasal intermittent positive pressure ventilation as postextubation support for preterm neonates in China: a randomized clinical trial. JAMA Pediatr. 2022;176(6):551-559. doi:10.1001/jamapediatrics.2022.0710 35467744 PMC9039831

[12] Brix N, Sellmer A, Jensen MS, Pedersen LV, Henriksen TB. Predictors for an unsuccessful INtubation-SURfactant-Extubation procedure: a cohort study. BMC Pediatr. 2014;14(1):155. doi:10.1186/1471-2431-14-155 24947477 PMC4072617

[13] Pezza L, Sartorius V, Loi B, . Evolution of ultrasound-assessed lung aeration and gas exchange in respiratory distress syndrome and transient tachypnea of the neonate. J Pediatr. 2023;256:44-52.e2. doi:10.1016/j.jpeds.2022.11.037 36493883

[14] Steinhorn R, Davis JM, Göpel W, ; International Neonatal Consortium. Chronic pulmonary insufficiency of prematurity: developing optimal endpoints for drug development. J Pediatr. 2017;191:15-21.e1. doi:10.1016/j.jpeds.2017.08.006 29173299

[15] De Luca D, Centorrino R. Nasal high-frequency ventilation. Clin Perinatol. 2021;48(4):761-782. doi:10.1016/j.clp.2021.07.006 34774208

[16] Juszczak E, Altman DG, Hopewell S, Schulz K. Reporting of multi-arm parallel-group randomized trials: extension of the CONSORT 2010 statement. JAMA. 2019;321(16):1610-1620. doi:10.1001/jama.2019.3087 31012939

[17] Restrepo RD, Hirst KR, Wittnebel L, Wettstein R. AARC clinical practice guideline: transcutaneous monitoring of carbon dioxide and oxygen—2012. Respir Care. 2012;57(11):1955-1962. doi:10.4187/respcare.02011 23107301

[18] Newth CJL, Venkataraman S, Willson DF, ; Eunice Shriver Kennedy National Institute of Child Health and Human Development Collaborative Pediatric Critical Care Research Network. Weaning and extubation readiness in pediatric patients. Pediatr Crit Care Med. 2009;10(1):1-11. doi:10.1097/PCC.0b013e318193724d 19057432 PMC2849975

[19] Dimitriou G, Greenough A, Endo A, Cherian S, Rafferty GF. Prediction of extubation failure in preterm infants. Arch Dis Child Fetal Neonatal Ed. 2002;86(1):F32-F35. doi:10.1136/fn.86.1.F32 11815545 PMC1721344

[20] Stevens B, Johnston C, Petryshen P, Taddio A. Premature Infant Pain Profile: development and initial validation. Clin J Pain. 1996;12(1):13-22. doi:10.1097/00002508-199603000-00004 8722730

[21] Fischer C, Bertelle V, Hohlfeld J, Forcada-Guex M, Stadelmann-Diaw C, Tolsa JF. Nasal trauma due to continuous positive airway pressure in neonates. Arch Dis Child Fetal Neonatal Ed. 2010;95(6):F447-F451. doi:10.1136/adc.2009.179416 20584802

[22] Brankovic M, Kardys I, Steyerberg EW, . Understanding of interaction (subgroup) analysis in clinical trials. Eur J Clin Invest. 2019;49(8):e13145. doi:10.1111/eci.13145 31135965

[23] European Medicines Agency. Guideline on the investigation of subgroups in confirmatory clinical trials. January 31, 2019. Accessed, March 8, 2023. https://www.ema.europa.eu/en/documents/scientific-guideline/guideline-investigation-subgroups-confirmatory-clinical-trials_en.pdf

[24] Wallace BC, Schmid CH, Lau J, Trikalinos TA. Meta-Analyst: software for meta-analysis of binary, continuous and diagnostic data. BMC Med Res Methodol. 2009;9:80. doi:10.1186/1471-2288-9-80 19961608 PMC2795760

[25] Chen L, Wang L, Ma J, Feng Z, Li J, Shi Y. Nasal high-frequency oscillatory ventilation in preterm infants with respiratory distress syndrome and ARDS after extubation: a randomized controlled trial. Chest. 2019;155(4):740-748. doi:10.1016/j.chest.2019.01.014 30955572

[26] Lemyre B, Davis PG, De Paoli AG, Kirpalani H. Nasal intermittent positive pressure ventilation (NIPPV) versus nasal continuous positive airway pressure (NCPAP) for preterm neonates after extubation. Cochrane Database Syst Rev. 2017;2(2):CD003212. doi:10.1002/14651858.CD003212.pub3 28146296 PMC6464652

[27] Walsh MC, Morris BH, Wrage LA, ; National Institutes of Child Health and Human Development Neonatal Research Network. Extremely low birthweight neonates with protracted ventilation: mortality and 18-month neurodevelopmental outcomes. J Pediatr. 2005;146(6):798-804. doi:10.1016/j.jpeds.2005.01.047 15973322

[28] Shalish W, Kanbar L, Kovacs L, . The impact of time interval between extubation and reintubation on death or bronchopulmonary dysplasia in extremely preterm infants. J Pediatr. 2019;205:70-76.e2. doi:10.1016/j.jpeds.2018.09.062 30404739

[29] Dell’Orto V, Bourgeois-Nicolaos N, Rouard C, . Cell count analysis from nonbronchoscopic bronchoalveolar lavage in preterm infants. J Pediatr. 2018;200:30-37.e2. doi:10.1016/j.jpeds.2018.04.074 29793870

[30] De Luca D, Dell’Orto V. Non-invasive high-frequency oscillatory ventilation in neonates: review of physiology, biology and clinical data. Arch Dis Child Fetal Neonatal Ed. 2016;101(6):F565-F570. doi:10.1136/archdischild-2016-310664 27354382

[31] Buzzella B, Claure N, D’Ugard C, Bancalari E. A randomized controlled trial of two nasal continuous positive airway pressure levels after extubation in preterm infants. J Pediatr. 2014;164(1):46-51. doi:10.1016/j.jpeds.2013.08.040 24094879

[32] De Luca D, Shankar-Aguilera S, Centorrino R, Fortas F, Yousef N, Carnielli VP. Less invasive surfactant administration: a word of caution. Lancet Child Adolesc Health. 2020;4(4):331-340. doi:10.1016/S2352-4642(19)30405-5 32014122

[33] Dell’Orto V, Raschetti R, Centorrino R, . Short- and long-term respiratory outcomes in neonates with ventilator-associated pneumonia. Pediatr Pulmonol. 2019;54(12):1982-1988. doi:10.1002/ppul.24487 31456358

[34] Cools F, Offringa M, Askie LM. Elective high frequency oscillatory ventilation versus conventional ventilation for acute pulmonary dysfunction in preterm infants. Cochrane Database Syst Rev. 2015;(3):CD000104. doi:10.1002/14651858.CD000104.pub4 25785789 PMC10711725

[35] Rüegger CM, Lorenz L, Kamlin COF, . The effect of noninvasive high-frequency oscillatory ventilation on desaturations and bradycardia in very preterm infants: a randomized crossover trial. J Pediatr. 2018;201:269-273.e2. doi:10.1016/j.jpeds.2018.05.029 29954606

[36] Tang S, Zhao J, Shen J, Hu Z, Shi Y. Nasal intermittent positive pressure ventilation versus nasal continuous positive airway pressure in neonates: a systematic review and meta-analysis. Indian Pediatr. 2013;50(4):371-376. doi:10.1007/s13312-013-0122-0 23255684

[37] Lemyre B, Davis PG, de Paoli AG. Nasal intermittent positive pressure ventilation (NIPPV) versus nasal continuous positive airway pressure (NCPAP) for apnea of prematurity. Cochrane Database Syst Rev. 2002;(1):CD002272. doi:10.1002/14651858.CD00227211869635

[38] Centorrino R, Loi B, De Luca D. Extremely preterm infants experienced good comfort with various nasal respiratory support techniques delivered with masks. Acta Paediatr. 2021;110(10):2753-2755. doi:10.1111/apa.15907 33964040

[39] Jensen EA, Roberts RS, Schmidt B. Drugs to prevent bronchopulmonary dysplasia: effect of baseline risk on the number needed to treat. J Pediatr. 2020;222:244-247. doi:10.1016/j.jpeds.2020.01.070 32143932

