# Supplementary material for: Effectiveness of Nasal Continuous Positive Airway Pressure vs Nasal Intermittent Positive Pressure Ventilation vs Noninvasive High-Frequency Oscillatory Ventilation as Support After Extubation of Neonates Born Extremely Preterm or With More Severe Respiratory Failure: A Secondary Analysis of a Randomized Clinical Trial
Source: JAMA Netw Open. 2023 Jul 3;6(7):e2321644. doi: 10.1001/jamanetworkopen.2023.21644 (PMC10318479; doi:10.1001/jamanetworkopen.2023.21644)
Supplement: Supplement 1. — Trial Protocol [file jamanetwopen-e2321644-s001.pdf]

Protocol title:

Noninvasive high frequency oscillation ventilation (NHFOV) *vs* continuous positive airway pressure (CPAP) *vs* noninvasive positive pressure ventilation (NIPPV) as post-extubation respiratory support in preterm neonates: a multicenter randomized trial.

Protocol identifying number: Clinical Trials.gov NCT02570217

Local number: N.3.0 (last version)/ date: Nov 15/2017

Principal Investigator: Prof. Yuan Shi

Children's Hospital of Chongqing Medical University, Ministry of Education Key Laboratory of Child Development and Disorders, Key Laboratory of Pediatrics, Chongqing, China

BACKGROUND

Respiratory distress syndrome (RDS) is the main cause of respiratory failure in preterm neonates, its incidence varying from ≈80% to ≈25% depending on gestational age.[1] When optimal prenatal care is provided, the best approach to treat RDS, according to several recent trials,[2,3] consists in providing continuous positive airway pressure (CPAP) from the first minutes of life using short binasal prongs or masks,[4,5] followed by early selective surfactant administration for babies with worsening oxygenation and/or increasing work of breathing. Any effort should be done to minimize the time under invasive mechanical ventilation (IMV).[6] Nonetheless, clinical trials have shown that a relevant proportion of preterm neonates fails this approach and eventually need IMV.[7,8,9] The duration of IMV is a well known risk factor for the development of broncho-pulmonary dysplasia (BPD) - a condition associated with significant morbidity and mortality.[10,11]

To minimize the duration of IMV, various non invasive respiratory support modalities are available in neonatal intensive care units (NICU). CPAP is presently the most common technique used in this regard. However, a systematic review has shown that non-invasive positive pressure ventilation (NIPPV) reduces the need for IMV (within one week from extubation) more effectively than NCPAP, although it is not clear if NIPPV may reduce need for intubation longterm and it seems to have no effect on BPD and mortality.[12] NIPPV main drawback is the

lack of synchronization, which is difficult to be accurately achieved and is usually unavailable. A more recent alternative technique is non-invasive high frequency oscillatory ventilation (NHFOV) which consists on the application of a bias flow generating a continuous distending positive pressure with oscillations superimposed on spontaneous tidal breathing with no need for synchronization. The physiological, biological and clinical details about NHFOV have been described elsewhere.[13]

To date, there is only one small observational uncontrolled study about the use of NHFOV after extubation in preterm infants.[14] Other relatively small case series or retrospective cohort studies suggested safety, feasibility and possible usefulness of NHFOV and have been reviewed elsewhere.[13] The only randomized trial published so far compared NHFOV to biphasic CPAP, in babies failing CPAP[15] and it has been criticized for methodological flaws and for not taking into account respiratory physiology.[16] An European survey showed that, despite the absence of large randomized clinical trials, NHFOV is quite widely used, at least in some Countries and no major side effects are reported, although large data about NHFOV safety are lacking.[17] This may be due to the relative NHFOV easiness of use but evidence-based and physiology-driven data are warranted about this technique.

NHFOV should theoretically provide the advantages of invasive high frequency oscillatory ventilation (no need for synchronization, high

efficiency in CO_2 removal, less volume/barotrauma) and nasal CPAP (non-invasive interface, oxygenation improvement by the increase in functional residual capacity through alveolar recruitment). NHFOV should allow to increase mean airway pressure (Paw) avoiding gas trapping and hypercarbia, thanks to the superimposed high frequency oscillations. Therefore, NHFOV is more likely to be beneficial for those neonates requiring high distending pressure to open up their lungs, such as babies at high risk of extubation failure due to severity of their lung disease. This may also be the case of extremely preterm, BPD-developing neonates who have increased airway resistances, while they are subjected to a deranged alveolarization and lung growth. Neonates presenting with respiratory acidosis may also benefit from NHFOV. Several animal and bench studies investigated the physiology and peculiarities of NHFOV[13] and these data should be used to conduct a physiology-guided trial in order to avoid errors done in the early trials about invasive high frequency ventilation.[16]

This study will be the first large trial aiming to compare CPAP *vs* NIPPV *vs* NHFOV in preterm neonates after surfactant replacement and during their entire NICU stay, to reduce the total need of invasive ventilation. Since there is a lack of formal data regarding NHFOV safety, some safety outcomes will also be considered. Specific subgroup analysis will be conducted for pre-specified groups of patients who may most likely benefit from NHFOV, according to the above-described

physiological characteristics.

OBJECTIVES

To test whether NHFOV is more efficacious than CPAP or NIPPV, as post-extubation respiratory support, to reduce the need for IMV all along their NICU stay in neonates born between 25 and 32-weeks' gestation.

INTENTIONALLY LEFT BLANK

METHODS

1.Trial design

This will be a **blinded, multi-center, three-arms, parallel, randomized, controlled trial with a superiority design,** conducted in China. Since safety data will also be analyzed it may be considered a **phase II/III** trial. Since the trial will enroll all eligible patients irrespective of their lung mechanics/physiopathology and eligibility will be judged on the basis of simple clinical data commonly used in NICU daily care, it may be considered a **pragmatic** trial.[18] Conversely, since subgroup analyses will be performed on patients defined according to their actual lung physiopathology, they should be considered **explanatory subgroup analyses**.[18] Results of subgroup analyses will anyway need confirmation in future, specifically designed trials. A total of 69 NICUs are included in this trial (**Fig.1 and appendix**). All these NICUs belong to 30 provinces or cities or autonomous regions of Chinese mainland (apart from Tibet which has been excluded for the too high altitude). The trial has been designed with the collaboration of international investigators experts in NHFOV and noninvasive respiratory support composing the international advisory board.

Figure 1. Neonatal Intensive Care Units participating to the trial.

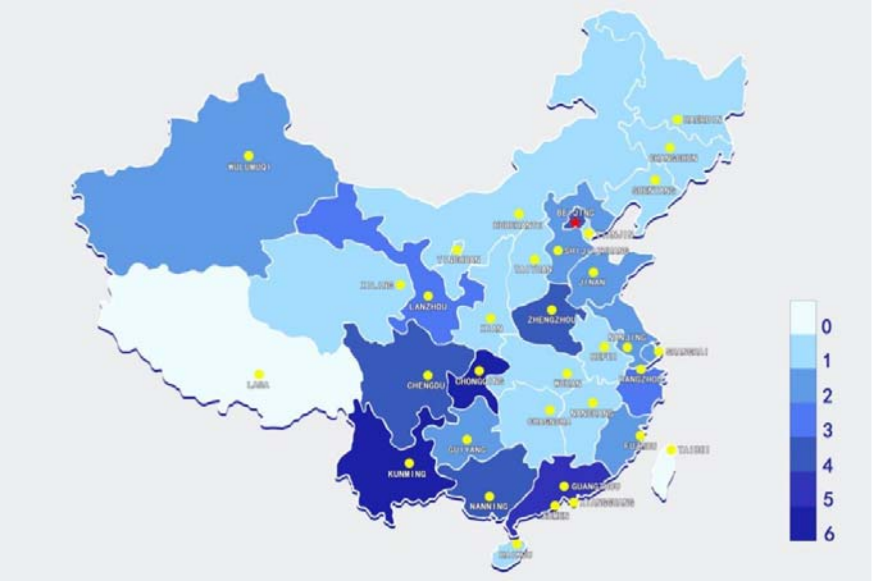

2. Inclusion criteria

For a neonate to be included three criteria must be fulfilled: **(1)** gestational age (GA) between 25+0 and 32+6 weeks (estimated on the postmenstrual date and early gestation ultrasonographic findings) and post-conceptional age <36 weeks; **(2)** assisted with any type of endotracheal ventilation; **(3)** ready to be extubated for the first time (extubation readiness requires fulfilling of all the following criteria: **a.** Having received at least one loading dose of 20 mg/kg and 5 mg/kg daily maintenance dose of caffeine citrate; **b.** pH>7.20 $PaCO_2$ ≤60 mmHg (these may be evaluated by arterialized capillary blood gas analysis or appropriately calibrated transcutaneous monitors[19] – see appendix. Venous blood gas values cannot be used); **c.** Paw ≤7-9

cmH_2O;[6] **d.** $FiO_2 \leq 0.30$; **e.** sufficient spontaneous breathing effort, as per clinical evaluation[20]).

3. Exclusion criteria

Neonates who never needed intubation and IMV are not eligible for the study; similarly, a neonate randomized but never extubated is not eligible in the study. Moreover, neonates with at least one of the following criteria are also not eligible: (1) major congenital anomalies or chromosomal abnormalities; (2) neuromuscular diseases; (3) upper respiratory tract abnormalities; (4) need for surgery known before the the first extubation; (5) Grade IV-intraventricular haemorrhage (IVH) occurring before the first extubation;[21] (6) congenital lung diseases or malformations or pulmonary hypoplasia; (7) birth weight <600 grams.

4. Randomization

Neonates will be randomized and assigned either to CPAP, NIPPV or NHFOV arms with a 1:1:1 ratio, when patients fulfil all inclusion criteria and extubation is deemed imminent (anyway within 1h). Randomization cannot be done earlier. Simple randomization will be done according to a computer-generated random number table and will be posted in a specific secured website available on 24/7. Twins will all be allocated in the same treatment group. **Infants randomized to one arm cannot crossover to the other or vice-versa during the study. Patients will remain under the**

assigned respiratory support until the weaning criteria (see below) will be met. In case of intubation, when the baby will be extubated, he will receive again his original treatment according to randomization. This can be summarized by **Fig.2.**

Figure 2. Study design. Neonates will stay on the assigned intervention up to the final weaning. No cross-over allowed. In case of intubation, when the baby will be extubated, he will receive again his original treatment according to randomization.

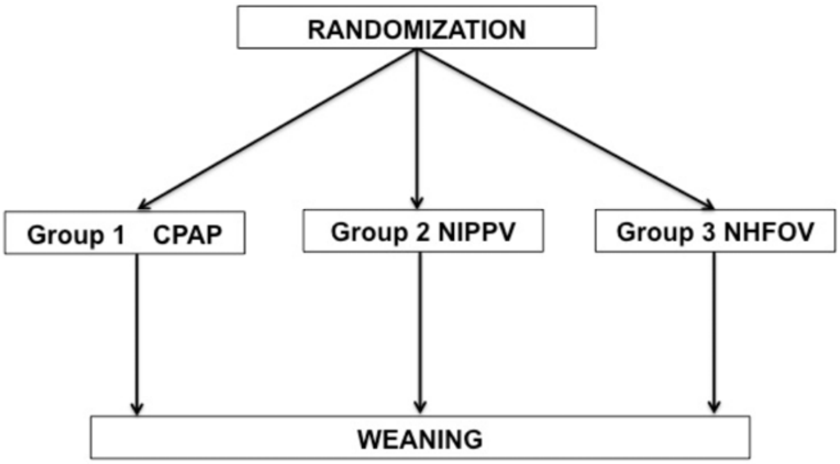

5. Blinding

Blinding towards the caregivers is impossible and blinding towards the patients makes no sense. **However, outcomes' assessors will be blinded, as endpoints will be recorded by investigators not involved in patients' care. An assessor per each participating NICU will be nominated. Moreover, investigators**

performing the final statistical analyses will be blinded to the treatment allocation, as data collected by assessors will be inserted in the dedicated website and the arms' allocation will be re-coded.

6. Primary outcomes

The primary outcomes will be: **(1) duration of IMV (in days); (2) ventilator-free days** (calculated as described in the appendix); **(3) the number of reintubation**. Neonates will be re-intubated if one of the following occurs:

a. severe respiratory acidosis (defined as $PaCO_2$>65 mmHg with pH<7.2);

b. hypoxia refractory to study intervention (defined as SpO_2< 90%, with FiO_2=0.4 and maximal pressures allowed in the study arm – see below) for at least 4h;

c. severe apnoea (defined as recurrent apnoea with >3 episodes/h associated with heart rate <100/min or a single episode of apnoea requiring bag and mask ventilation, or associated with SpO_2<85% and FiO_2>0.6);

d. pulmonary haemorrhage (defined as brightly blood tracheal secretion associated with sharp increase in oxygen and Paw requirement and with occurrence of "white lungs", new infiltrates or consolidations at the chest X-rays);

e. severe respiratory distress (defined as Silverman score >4) for at least 4h;

f. haemodynamic instability, defined as mean arterial pressure <10th percentile of appropriate nomograms[22,23] or anyway need of dopamine (if >5 γ/Kg/min) or dobutamine (if >5 γ/Kg/min) or any dose of noradrenaline, adrenaline, milrinone, nitric oxide or other pulmonary vasodilators.

g. cardio-respiratory arrest.

7. Secondary outcomes

The secondary outcomes will be: **(1) airleaks** (pneumothorax and/or pneumomediastinum) occurred *after* the extubation; **(2) BPD**, defined according to the NICHD definition (more details in the appendix);[24] **(3) haemodynamically significant patent *ductus arteriosus* (PDA),** defined according to local NICU protocols; **(4) retinopathy of prematurity (ROP) > 2nd stage**;[25] **(5) necrotizing enterocolitis (NEC) ≥ 2nd stage**;[26] **(6) IVH>2nd grade**;[21] **(7) need for postnatal steroids; (8) in-hospital mortality**; **(9) composite mortality/BPD**; **(10) Weekly weight gain (in grams/day) for the first 4 weeks of life or until NICU discharge, whichever comes first.**

8. Safety outcomes

The safety outcomes will be the following: **(1) weekly number of vomiting** per day; **(2) weekly volume of gastric residual** (ml/day); **(3) weekly number of apnoeas** per day; **(4) nasal skin injury** (weekly

defined by a clinical score[27] as: 0 (zero, absence of injury), stage I (non-blanching erythema), stage II (superficial erosion), stage III (necrosis of full thickness of skin) – more details in the appendix). **These outcomes will be averaged over each week for the first 4 weeks of life or until NICU discharge, whichever comes first.** Finally, **(5) Premature Infant Pain Profile score[28]** will be considered (averaged from values available in the first 48h from the allocation to CPAP, NIPPV or NHFOV).

9. Standard protocol approvals, registrations, and patient consents

The study was approved by the Ethics Committee of Daping Hospital (n.201721) and registered in the clinicaltrial.gov registry (ID: NCT03181958). The trial was performed in accordance with the approved guidelines and regulations of the participating institutions. Informed consent will be obtained antenatally or upon NICU admission from parents or guardians.

10. Study Intervention

When the neonate had fulfilled the extubation criteria, this latter will took place with a gentle intratracheal suction, following local policies. Upper airways will then be suctioned and intervention will be started immediately as follows:

10.1 Ventilators

- **CPAP**: CPAP will be provided by either variable flow or continuous

flow devices, as there is no evidence that one type of CPAP generator would be better than any other.[29]

- **NIPPV**: NIPPV will be provided by any type of neonatal ventilator. Synchronization will not be applied, as many currently marketed neonatal ventilators usually do not provide it for NIPPV.[30]
- **NHFOV**: NHFOV will only be provided with piston/membrane oscillators able to provide a real oscillatory pressure with active expiratory phase (that is, Acutronic FABIAN-III, SLE 5000, Loweinstein Med LEONI+, Sensormedics 3100A). Other machines providing high frequency ventilations will not be used.

Before the beginning of the study all ventilators will be checked to ensure that there is no malfunction.

10.2 Interfaces

CPAP, NIPPV and NHFOV will be all administered through short, low-resistance binasal prongs and/or nasal masks, since these are supposed to be the best in terms of resistive charge, leaks and/of comfort.[4,5] Nasal prongs size will be chosen according to the nares' diameter as the best fitting ones (the largest ones that fit the nares without blanching the surrounding tissues) and following manufacturer's recommendations. Nasal masks will also be appropriately sized according to manufacturer's recommendations. Alternating masks and prongs, according to clinical evaluation, is allowed in order to reduce the risk for nasal skin injury. Particular care

(e.g.: pacifiers, positioning, nursing) will be applied to reduce leaks and improve patients' comfort. These latters will be evaluated through a dedicated 30 min observation period when study intervention will be instigated. Non pharmacological sedation with pacifier and 33% glucose solution will be provided, when needed; no other sedation will be allowed. RAMCannula® are not allowed in the trial due to their resistive charge and their relevant pressure leaks.[31,32]

10.3 Ventilatory management

The three different respiratory supports will be managed as follows:

- **CPAP**: Neonates assigned to the CPAP group were initiated on a pressure of 5 cmH_2O. CPAP can be raised in steps of 1 cmH_2O up to 8 cmH_2O. If this is not enough to maintain SpO_2 between 90% and 95%, FiO_2 will be added up to 0.40.
- **NIPPV**: neonates assigned to the NIPPV group will be started with the following parameters: **a)** positive end-expiratory pressure (PEEP) of 4 cmH_2O (can be raised in steps of 1 cmH_2O to max 8 cmH_2O, according to the oxygenation). **b)** Peak Inspiratory Pressure (PIP) of 15 cmH_2O (can be raised in steps of 1 cmH_2O to max 25 cmH_2O, according to oxygenation, $PaCO_2$ levels and the chest expansion); maximal allowed FiO_2 will be 0.40 and SpO_2 targets will be 90-95%. **c)** inspiratory time (IT) will be 0.45 – 0.5 sec (according to clinicians' evaluation of leaks and the appearance of the pressure curve: a small pressure plateau is required and flow

may be set accordingly) and rate will be started at 30 bpm (can be raised in steps of 5 bpm to max 50 bpm, according to $PaCO_2$ levels).

- **NHFOV**: neonates assigned to NHFOV will be started with the following boundaries:

a) Paw of 10 cmH_2O (can be changed in steps of 1 cmH_2O within the range range 5-16 cmH_2O); Paw will be titrated (within the range) according to open lung strategy, performing alveolar recruitment, similar to what is done in endotracheal high frequency oscillatory ventilation (see **Fig.3**) targeting a $FiO_2 \leq 25\text{-}30\%$.[33] Maximal allowed FiO_2 will be 0.40 and SpO_2 targets will be 90%-95%. **b)** frequency of 10Hz (can be changed in steps of 1Hz within the range 8-12Hz). **c)** Inspiratory time 50% (1:1).[34] **d)** amplitude 25 cmH_2O (can be changed in steps of 5 cmH_2O within the range 25-50 cmH_2O);[34,35] amplitude will be titrated according to $PaCO_2$. It is not strictly necessary to have visible chest oscillations, as $PaCO_2$ elimination during NHFOV also occurs in the upper airway dead space.[36] In case of hypercarbia, amplitude will be increased first and then frequency will be lowered (within the above-described ranges), however, if nasal masks are used amplitude should be kept at the maximum and $PaCO_2$ controlled by frequency titration, as the CO_2 elimination using nasal masks seems inferior.[37]

INTENTIONALLY LEFT BLANK

Figure 3. Alveolar recruitment according to the open lung strategy, as described in[33]. This should be followed in NHFOV arm, and repeated as per clinical need within the suggested mean airway pressure boundaries (5-16 cmH_2O, with changes in steps of 1 cmH_2O). [*Courtesy of Prof. A. van Kaam and Prof. D. De Luca*].

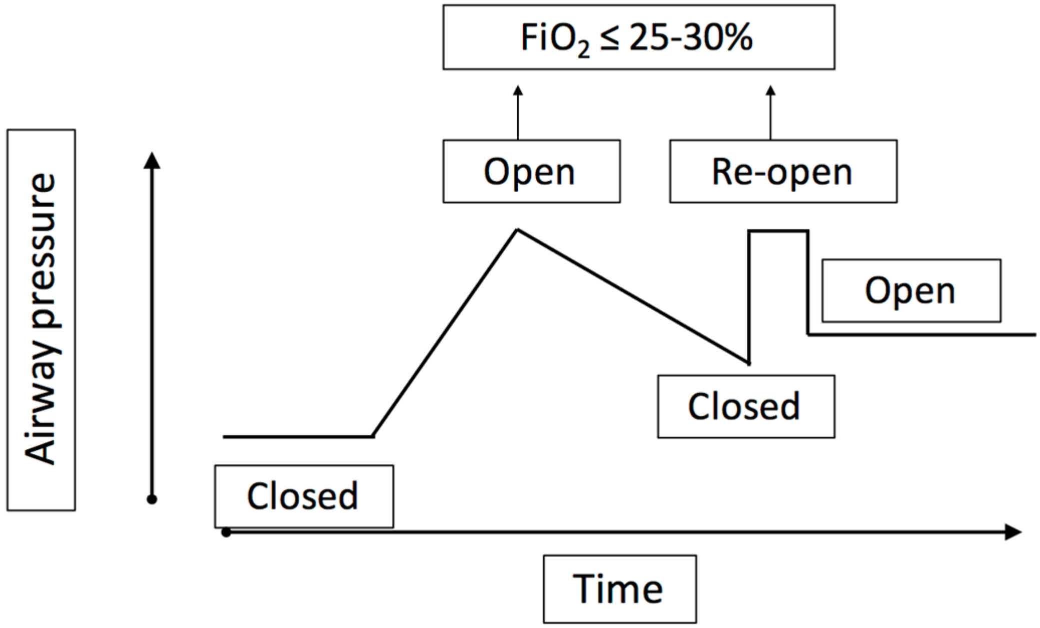

10.4 Monitoring and concurrent treatments/diagnostic measures

$PaCO_2$ will be monitored using arterialized capillary blood gas analysis and/or transcutaneous monitors according to local policies. Transcutaneous monitoring will be performed according to the American Association of Respiratory Care guidelines[19] and the

manufacturer's recommendations. Frequency of blood gas analysis will be decided by the attending clinicians. All neonates will be continuously monitored for SpO_2, ECG, heart and respiratory rate. To avoid abdominal distention, a feeding tube will be placed in the stomach through the mouth and gas will periodically aspirated according to nurses' evaluation in all study arms.

Moreover, the following treatments or tests will be provided:

- Heart ultrasound to evaluate cardiac morphology, pulmonary pressures and PDA, within the first 3 days of life and subsequently repeated, if needed.
- Cerebral ultrasound within 48 hours of life and weekly thereafter, until discharge, if needed.
- Routine measures to prevent BPD; routine fluid/nutritional policy; routine caffeine therapy.
- Placement of umbilical central venous catheter and/or peripherally inserted central venous lines. Placement of arterial lines if needed, according to local policies.
- Routine therapies according to local policies (i.e.: antibiotics, PDA closure drugs…).

In general, routine clinical assistance and nursing will not be changed because of the study, out of the trial intervention; the clinical assistance will be identical in the three study arms. No additional blood samples are required for this study.

10.5 Weaning from study interventions

The study intervention will be progressively weaned, according to clinical evaluation and respecting the following guidelines:

- in the CPAP arm, pressure will be reduced by 1 cmH_2O steps down to a minimum of 3 cmH_2O;
- in the NIPPV arm, PIP and PEEP will be reduced by 1 cmH_2O steps down to a minimum of 3 and 5 cmH_2O, respectively. Similarly, frequency will be reduced to a minimum level of 20 bpm in steps of 5 bpm.
- in the NHFOV arm, amplitude will be reduced to the minimum initial level of 20 cmH_2O and Paw will be reduced by 1 cmH_2O down to a minimum of 3 cmH_2O.

The study intervention (CPAP, NIPPV or NHFOV) will be stopped when the above-described minimum parameters are reached and maintained for at least 48h with the following: (1) FiO_2 ≤0.25; (2) Silverman score <3; (3) no apnoeas or bradycardia without spontaneous recovery. If a baby will desaturate (SpO_2 <85% with FiO_2>25%) or has relevant dyspnoea (Silverman≧3) or more than 3 apnoeas/day the intervention (CPAP, NIPPV or NHFOV) will be restarted for at least 48h and then re-evaluated. The end of study intervention may occur at any time during hospitalization if the above described criteria are met. When study interventions end, the neonate

may be placed under low flow oxygen therapy (max 1 L/min), if needed, according to clinicians' evaluation and local policies. Anyway, when a post-conceptional age of 36 weeks is reached, if the patient still needs noninvasive respiratory support, he/she will be shifted to CPAP and managed according to clinical evaluation and local policies.

11. End of the study

A patient may exit from the study for any of the following reasons:

1. Death.
2. In any case, when the 36 weeks' post-conceptional age is reached.
3. If parents or guardians withdraw an already given consent for the participation (in that case the patient will keep receiving the whole routine clinical assistance; data acquired up to that point will be immediately destroyed).

12. Sample size calculation

It is difficult to calculate a sample size, since this is the first to investigate CPAP *vs* NIPPV *vs* NHFOV in post-extubation phase in preterm babies. However, a previous prospective, cohort, non-randomized, pilot study comparing post-extubation NIPPV and NHFOV in preterm neonates provides data about the primary outcome "duration of mechanical ventilation". This study showed a reduction of ≈30% for babies receiving NHFOV, as compared to those treated with NIPPV.[38]A randomized trial of NIPPV vs CPAP by Ramanathan et al. showed a similar reduction.[39] Since these trials have not the same

design of ours, we decide to be more prudent and we aimed a difference of 20% in the duration of mechanical ventilation. Considering an alpha-error of 0.05 (with a Bonferroni correction at 0.017) and a power of 95%, 480 neonates should be enrolled in each arm (with a 1:1:1 design). Thus, a total of at least 1440 neonates will be enrolled. Sample size calculation has been performed with GPower 3.1.9.3.[40]

13. Data collection

All data for trial analysis are routine clinical items that can be obtained from the clinical notes. Data will be recorded in real time (every day) on web-based case report forms provided by OpenCDMS. The website will be tested with fictitious data before the actual enrolment. Data will be entered by an assessor per each center. Assessors will be research nurses or local investigators blinded to the study intervention, as they are not involved in patients' care. Access to the form will be password protected and participants will be identified by trial number only. Clinical information will be collected at the following time-points:

Before the intervention begins: information on eligibility; baseline clinical informations, respiratory diagnosis, critical risk index for babies-II (CRIB-II) score.[41]

Following study intervention: ventilator parameters, SpO_2, blood gas values before the extubation if available (it is suitable to have at least transcutaneous blood gas values). PaO_2, $PaCO_2$, SpO_2 and pH between

6h and 24h from the extubation.

Follow-up: NICU length stay, duration of IMV, number of reintubation, ventilator free days, duration of oxygen therapy, duration of the study intervention (CPAP, NIPPV or NHFOV), airleaks, PDA, BPD, ROP >2nd stage, NEC≥2nd stage, IVH>2nd grade, need for postnatal steroids, in-hospital mortality, composite mortality/BPD, weekly weight gain (in grams/day) for the first 4 weeks of life or until NICU discharge, whichever comes first. Moreover, the following safety data will be recorded: weekly number of vomiting per day, weekly volume of gastric residual (ml/day); weekly number of apnoeas per day; nasal skin injury (weekly defined by a 1-2-3 clinical score[24] – more details in the appendix). These outcomes will be averaged over each week for the first 4 weeks of life or until NICU discharge, whichever comes first. Finally, the Premature Infant Pain Profile score[25] recorded in the first 48h from the allocation will be recorded. Abdominal circumference at 48h and 96h from the instigation of CPAP, NIPPV or NHFOV will also be recorded.

14. Statistics

Data analysis will be performed blindly to the type of treatment received. An *intention-to-treat* analysis will be applied. An *interim* analysis will be performed at 50% of the enrolment. First, data will be checked for normality using Kolmogorov-Smirnov test and results will be presented as odds ratio (OR) and 95% confidence interval (CI) or adjusted OR and 95%CI, and mean ± standard deviation or median

(quartiles), as appropriate. ANOVA and Mann-Whitney test will be applied, according to data distribution. Proportions will be tested using Chi^2 or Fisher tests, as appropriate.

If required, according to type of variable and their distributions, logistic or linear regressions will be performed. Multivariate regressions will also be performed for selected outcomes if needed (that is, if a baseline characteristic of the enrolled population differs between the two arms with a $p<0.2$ at the univariate analysis, then the results will be adjusted for that variable). In that case analysis of multicollinearity will be previously performed considering condition index of Eigenvalues and variables inserted in the model will have to carry a Variance Inflation Factor <2.[42,43] *p*-values <0.05 will be considered statistically significant.

The following sub-group analysis will be performed:

- **1.** Subgroup analysis for **babies ≤28 weeks' gestation**.
- **2.** Subgroup analysis for **babies who have been invasively ventilated for at least 1 week from birth.**
- **3.** Subgroup analysis for babies with **$PaCO_2$>50mmHg before the extubation or at the 6h or 24h after extubation**.

15. Data Monitoring Board (DMB)

Dr. Dezhi Mu, Professor of Pediatrics, West China Second University Hospital, Sichuan University, Chengdu, Sichuan.

Dr. Mingyan Hei, Professor of Neonatal Centre, Beijing Children's Hospital, Capital Medical University, Beijing National Centre for

Children's Health, Beijing, China.

Dr. Lyv Deliang, Shenzhen Centre for chronic disease control.

Dr. Lyv Deliang served as a consultant for the statistical analyse

16. International advisory panel

International colleagues were consulted for the protocol preparation, the training on trialed respiratory support techniques and technical problems that may arise during the study. They will also be able to analyze data in the *interim* analysis at approximately 50% of the trial enrolment. The panel will be composed by international neonatologists or pediatric intensivists experts in respiratory care. This is unusual in a trial about neonatal ventilation but will help to increase the quality of data. The board will advice the principal investigator (YS) who will remain the only complete responsible for any aspect of the trial.

INTENTIONALLY LEFT BLANK

ETHICAL CONSIDERATIONS

This trial is worth to be conducted given the uncertainty about the effectiveness of the different respiratory support techniques tested. Moreover, NHFOV might be actually superior to the other techniques, as we may hypothesize from the currently available data. In detail, many different types of noninvasive respiratory support are now available and we do not know yet which is the best policy to be applied especially in the babies at highest risk, (i.e.: in those developing BPD). NHFOV has been already studied in preliminary cross-over trials, in bench and animal studies,[13] while invasive HFOV is routinely used for severe respiratory failure worldwide. NIPPV has been studied in several randomized controlled trials enrolling smaller population and/or without triple comparison against CPAP and NHFOV. Thus, there is actually a great drive towards non-invasive ventilation, especially for preterm neonates, and this study is a new step within this framework.

Thus, the risks for babies are minimized and the monitoring will report quickly any problem, if any. Out of the studied intervention, the participation to the study will not change in any way the routine clinical assistance set for every patient. Data will be recorded anonymously and will be secured and accessible only to the investigators and to the parents/guardians of the enrolled patients. Data of a specific patient will be immediately destroyed if an already given consent is withdrawn. In no case the recorded data will be used for purposes out of those specified in the trial protocol. Moreover, the trial is only funded by a public Chinese research program, thus it will not have external industrial

influences and has the merit to try filling the lack of public funding for neonatal ventilation trials.[44]

INTENTIONALLY LEFT BLANK

APPENDIX

A1. CLARIFICATIONS FOR EXCLUSION CRITERIA

Neonates who never needed intubation and IMV are not eligible for the study; similarly, a neonate randomized but never extubated is not eligible in the study. This means that, if a randomized neonate is not actually extubated within 1 hour because there has been a worsening of his conditions or death, he is excluded the study. **Randomization must be done as close as possible to the extubation, once all inclusion criteria are fulfilled (see above), and anyway within 1h from the actual extubation.**

1. **Some exclusion criteria are represented by congenital disorders: when a patient is affected by these disorders his biology and physiology are significantly changed and are not eligible for the study and will not be randomized. If the condition has been discovered/suspected after the randomization but before the study inclusion (that is, before extubation), they will not receive the study intervention and will not entry in the study. If one of these conditions is diagnosed after the inclusion in the study, the neonate will be excluded *a posteriori***. This is the case of neonates with major congenital anomalies, chromosomal abnormalities, neuromuscular diseases, congenital upper respiratory tract abnormalities and congenital lung diseases or malformations or hypoplasia. Examples of these conditions are: genetic syndromes, surfactant protein

defects, congenital adenomatous pulmonary malformations, congenital diaphragmatic hernia or sequestration, congenital hypoventilation syndrome, pulmonary hypoplasia or any metabolic disease.

2. **Same applies for the need for surgery anticipated antenatally or before the first extubation, as this is usually related to congenital malformations. These neonates are not eligible for the study and will not be randomized. If the condition has been discovered/suspected after the randomization but before the study inclusion (that is, before extubation) they will not receive the study intervention and will not entry in the study. If a surgery will be needed later during the NICU hospitalization for other reasons (for instance for PDA ligation or NEC), the patient will regularly continue the trial.** Conditions needing surgery will be noticed in the web-based database.
3. **Grade IV-IVH known before the first extubation is a significant risk factor for prognosis and for quality of life. Continuing the NICU care in this situation may be considered unethical, depending to different local settings, cultures, ethical and religious beliefs. This may significantly impact on the trial outcomes. These neonates are not eligible for the study and will not be randomized. If grade IV-IVH has been discovered/suspected after the randomization but before the study inclusion (that is, before extubation), they will not receive the study intervention and will not entry in the study.** If Grade IV-IVH will be diagnosed after the study inclusion, the patient

will continue the study regularly and this will be noticed amongst the outcomes.

A2. LIST OF STUDY DEFINITION/ASSESSMENTS (IN ALPHABETICAL ORDER)

- **Antenatal steroids.** Antenatal steroid prophylaxis will be considered complete if two 12 mg-doses of betamethasone 24h apart and between 1 day and 7 days before the delivery had been given.
- **Blood gas analysis.** Blood gas values may only be obtained in following three ways (venous blood gas analysis is not allowed in the study).

Arterial blood from indwelling arterial lines, if one of these was placed for clinical reasons. As these are likely to be unavailable in the majority of cases, the following two alternative techniques may be used.

Arterialized capillary blood gas analysis is performed, warming a patient's heel for 10 minutes and collecting 200 μL of blood into a heparinized micro-tube. This must be analyzed by a blood gas analyzer within 5 minutes. Blood gas analysis will be obtained upon attending neonatologist decision.

Transcutaneous blood gas monitoring will be performed according to American Associations of Respiratory Care guidelines[19] and the device manufacturer's recommendations and using an electrode temperature of 44 °C for a short time (max 10-15min). Particular care must be provided to avoid skin injury in extremely preterm neonates: in some cases, a temperature of 42°C may be more suitable.

- **BPD definition as per NICHD definition (for neonates ≤32 weeks' gestation)[24]**

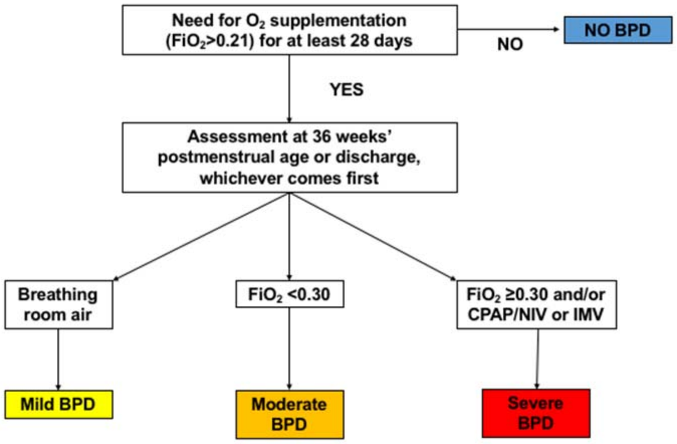

- **Clinical Risk Index for Babies (CRIB-II) score.[41]** This is an estimator of the clinical severity at the NICU admission. CRIB-II score considers 4 variables: *birth weight, GA, base excess within the 1st hour of life and temperature at the admission* (see below as reproduced from [41])). An online calculator is available at www.sfar.org/scores2/crib22.php

Birthweight (g) and gestation (weeks):

The maximum (worst) score for birthweight and gestation is 15, which is obtained for a 22 week male infant of less than 501 g birthweight

Male infants

Birthweight (g)	22	23	24	25	26	27	28	29	30	31	32
2751 to 3000											0
2501 to 2750										1	0
2251to 2500									3	0	0
2001to 2250									2	0	0
1751to 2000								3	1	0	0
1501to 1750						6	5	3	2	1	0
1251to 1500					8	6	5	3	3	2	1
1001to 1250		12	10	9	8	7	6	5	4	3	3
751 to 1000		12	11	10	8	7	7	6	6	6	6
501 to 750	14	13	12	11	10	9	8	8	8	8	
251 to 500	15	14	13	12	11	10	10				

Gestation (weeks)

Female infants

Birthweight (g)	22	23	24	25	26	27	28	29	30	31	32
2751 to 3000											0
2501 to 2750										1	0
2251 to 2500									2	0	0
2001 to 2250									1	0	0
1751 to 2000								3	1	0	0
1501 to 1750						6	4	3	1	0	0
1251 to 1500					7	5	4	3	2	1	1
1001 to 1250		11	10	8	7	6	5	4	3	3	3
751 to 1000		11	10	9	8	7	6	5	5	5	5
501 to 750	13	12	11	10	9	8	8	7	7	7	
251 to 500	14	13	12	11	10	10	10				

Gestation (weeks)

Temperature at admission (°C)

≤29·6	5
29·7 to 31·2	4
31·3 to 32·8	3
32·9 to 34·4	2
34·5 to 36	1
36·1 to 37·5	0
37·6 to 39·1	1
39·2 to 40·7	2
≥40·8	3

Base excess (mmol/L):

<−26	7
−26 to −23	6
−22 to −18	5
−17 to −13	4
−12 to −8	3
−7 to −3	2
−2 to 2	1
≥3	0

Sex, birthweight (g) and gestation (weeks): ______
Temperature at admission (°C): ______
Base excess (mmol/L): ______

Total CRIB II Score ☐

The logistic regression equation relating CRIB II to mortality (CRIB II algorithm) is:
Log odds of mortality = G = −6·476 + 0·450×CRIB II
Probability of mortality = exp(G)/[1+exp(G)]
The range of possible CRIB II scores is 0 to 27

Clinical risk index for babies II (CRIB II) score

- **Time on CPAP/NIPPV/NHFOV.** Number of days spent under these respiratory supports will be registered and rounded to the closest entire number.
- **Gestational age (GA).** GA is determined based on sure dates of last menstrual period or early ultrasound scan (within the first trimester). If a discrepancy of more than 2 weeks exists, the early ultrasound scan will be chosen.
- **Nasal injuries.** These are classified by using a clinical score[26] as stage I (non-blanching erythema), stage II (superficial erosion), stage III (necrosis of full thickness of skin) in the skin area in contact with nasal prongs (see below, as reproduced from[27]). The score will be 0 (zero), in case of absence of any injury.

INTENTIONALLY LEFT BLANK

- **Premature Infant Pain Profile (PIPP) score.[28] (see below, reproduced from [28])**

Table V – Premature Infant Pain Profile (PIPP)

Indicators	0	1	2	3
GA in weeks	≥ 36 weeks	32 to 35 weeks and 6 days	28 to 31 weeks and 6 days	< 28 weeks
Observe the NB for 15sec				
Alertness	Active<br>Awake<br>Opened eyes<br>Facial movements present	Quiet<br>Awake<br>Opened eyes<br>No facial movements	Active<br>Sleep<br>Closed eyes<br>Facial movements present	Quiet<br>Sleeping<br>Closed eyes<br>No facial movements
Record HR and SpO_2				
Maximal HR	↑ 0 to 4 bpm	↑ 5 to 14 bpm	↑ 15 to 24 bpm	↑ ≥ 25 bpm
Minimal Saturation	↓ 0 to 2.4%	↓ 2.5 to 4.9%	↓ 5 to 7.4%	↓ ≥ 7.5%
Observe NB for 30 sec				
Frowned forehead	Absent	Minimal	Moderate	Maximal
Eyes squeezed	Absent	Minimal	Moderate	Maximal
Nasolabial furrow	Absent	Minimal	Moderate	Maximal

Absent is defined as 0 to 9% of the observation time; minimal, 10% to 39% of the time; moderate, 40% to 69% of the time; and maximal as 70% or more of the observation time. In this scale, scores vary from zero to 21 points. Scores equal or lower than 6 indicate absence of pain or minimal pain; scores above 12 indicate the presence of moderate to severe pain.
GA – Gestational Age. NB – Newborn.

- **Pulmonary hypoplasia.** This will be clinically defined if anamnestic (prenatal findings: small lung volume), imaging (diffuse chest x-ray opacity or hypo-density) and clinical data (extremely low gestational age, olygo-anhydramnios, severe pulmonary hypertension) are present. Pulmonary hypoplasia usually does not allow survival.
- **Respiratory main diagnosis. A respiratory main diagnosis that required IMV (± surfactant administration) has to be given according to the following criteria. RDS:** respiratory distress appearing within the first 24 h of life, with complete, sustained, and prompt response to surfactant or lung recruitment or both; additional non-mandatory criteria are lung imaging (chest X-rays or ultrasound, according to local policies) supporting the diagnosis or lamellar body counts ≤30 000/mm³, or both.[45] **Pneumonia**: broncho-alveolar lavage fluid or blood positive culture or C-reactive protein and/or procalcitonin beyond the normal values, together with

radiological signs of infection (infiltrates and/or consolidation and/or loss of aeration).[46] **Sepsis (international pediatric sepsis definition)**: presence of systemic inflammatory response syndrome (SIRS) together with a suspected or proven (by positive culture, tissue stain, or polymerase chain reaction test) infection caused by any pathogen OR a clinical syndrome associated with a high probability of infection.[47] Evidence of SIRS is given by the presence of at least two of the following four criteria, one of which must be abnormal temperature or leukocytes:

- Core temperature >38.5°C or <36°C.
- Tachycardia, defined as a mean heart >180 bpm in the absence of external stimulus, chronic drugs, or painful stimuli; or otherwise unexplained persistent elevation over a 0.5- to 4-hr time period OR bradycardia, defined as a mean heart rate <100 bpm in the absence of external vagal stimulus, -blocker drugs, or congenital heart disease; or otherwise unexplained persistent depression over a 0.5-hr time period.
- Mean respiratory rate >60/min or need for IMV for an acute process not related to underlying neuromuscular disease or the receipt of general anesthesia.
- Leukocyte count elevated or depressed or 10% immature neutrophils.

Evidence of infection includes positive findings on anamnesis, clinical exam, imaging, or laboratory tests.[48] **Meconium aspiration syndrome (MAS):** presence meconium-stained amniotic fluid and secretions upon tracheal suctioning with onset of respiratory distress early from birth

and chest X-rays or lung ultrasound typical for MAS.[48] **Neonatal ARDS:** defined as per the international Montreux definition.[45]

- **Ventilator free days (VFD)** defined as the number of days spent in the NICU without IMV. One point is given for each day during the NICU stay that patients are both alive and free of mechanical ventilation; as death, is the worst outcome a dead patient is given zero VFD.[49]

INTENTIONALLY LEFT BLANK

A3. LIST OF DATA TO BE COLLECTED IN THE ELECTRONIC CRF

Basic patients' data

1. Sex (male/female).

2. Gestational age (weeks without decimals).

3. Birth Weight (g).

4. CRIB-II score[41] at the NICU admission (as described above or through the online calculator cited above).

5. Day of age at the first extubation (d).

6. Delivery type (Vaginal birth/Elective caesarean delivery/Emergency caesarean delivery).

7. Complete Antenatal steroids (yes/no – as defined above).

8. Apgar score at 1 and 5 min.

9. Surfactant given: (yes/no and which type of surfactant).

10. Surfactant dose (mg/kg).

11. Number of surfactant doses, if any (1,2,3).

12. Mean airway pressure (Paw) at the extubation (cmH_2O).

13. FiO_2 and SpO_2 before extubation (% - right before extubation).

14. PaO_2 before extubation (mmHg – obtained as described above from the closest available measurement).

15. $PaCO_2$ before extubation (mmHg – obtained as described above from the closest available measurement).

16. pH before extubation (if available, obtained as described above from the closest available measurement).

17. Lactate before extubation (if available, obtained as described above from the closest available measurement).

18. Type of ventilation at the extubation (Conventional/HFOV).

19. Main early respiratory diagnosis (RDS/Pneumonia/Sepsis/ARDS/MAS…).

20. Main reason for re-intubation (if any).

Outcomes

1. Extubation failure at 48h (yes/no).

2. Paw (cmH_2O), PaO_2 (mmHg), $PaCO_2$ (mmHg) and FiO_2 (%) between 6 and 24h after extubation.

3. Duration of invasive ventilation (d).

4. Ventilator free-days (calculated as described above).

5. Number of re-intubations during the NICU stay (total number).

6. Duration of treatment (CPAP, NIPPV or NHFOV – d).

7. Duration of oxygen treatment (d).

8. Need for postnatal steroids (yes/no).

9. In-hospital mortality (yes/no).

10. Weekly weight gain (g/d) (averaged over each week for the first 4 weeks of life or until NICU discharge, whichever comes first, as described above).

11. Air leaks after the extubation (yes/no).

12. BPD[23] (no/mild/moderate/severe – as described above).

13. haemodynamically significant PDA (diagnosed according to local

NICU protocols).

14. ROP >2nd stage.

15. NEC ≥2nd stage.

16. IVH >2nd grade.

17. NICU length of stay (d).

18. Weekly number of vomiting per day (averaged over each week for the first 4 weeks of life or until NICU discharge, whichever comes first, as described above).

19. Weekly volume of gastric residual (ml/d) (averaged over each week for the first 4 weeks of life or until NICU discharge, whichever comes first, as described above).

20. Weekly number of apnoeas per day (averaged over each week for the first 4 weeks of life or until NICU discharge, whichever comes first, as described above).

21. Premature Infant Pain Profile score (averaged from the values available in the first 48h from the allocation, as described above).

22. Nasal skin injury (weekly defined by a 0-1-2-3 clinical score, as described above).

23. Abdominal circumference (cm) at 48h and 96h after study intervention.

REFERENCES

1. Lemons JA, Bauer CR, Oh W, Korones SB, Papile LA, Stoll BJ, Verter J, Temprosa M, Wright LL, Ehrenkranz RA, Fanaroff AA, Stark A, Carlo W, Tyson JE, Donovan EF, Shankaran S, Stevenson DK. Very low birth weight infant outcomes of the NICHD neonatal research network. Pediatrics. 2001;107: 1–8.

2. Schmölzer GM, Kumar M, Pichler G, Aziz K, O'Reilly M, Cheung PY. Non-invasive versus invasive respiratory support in preterm infants at birth: systematic review and meta-analysis. BMJ. 2013;347:f5980.

3. Subramaniam P, Ho JJ, Davis PG. Prophylactic nasal continuous positive airway pressure for preventing morbidity and mortality in very preterm infants. Cochrane Database Syst Rev. 2016;6:CD001243.

4. De Paoli AG, Davis PG, Faber B, Morley CJ. Devices and pressure sources for administration of nasal continuous positive airway pressure (NCPAP) in preterm neonates.
Cochrane Database Syst Rev. 2008;1:CD002977.

5. Kieran EA, Twomey AR, Molloy EJ, Murphy JF, O'Donnell CP. Randomized trial of prongs or mask for nasal continuous positive airway pressure in preterm infants. Pediatrics. 2012;130:e1170-6.

6. Sweet DG, Carnielli V, Greisen G, Hallman M, Ozek E, Plavka R, Saugstad OD, Simeoni U, Speer CP, Vento M, Visser GH, Halliday HL. European Consensus Guidelines on the Management of Respiratory Distress Syndrome - 2016 Update. Neonatology. 2017;111:107-125.

7. Stefanescu BM, Murphy WP, Hansell BJ, Fuloria M, Morgan TM, Aschner JL. A randomized, controlled trial comparing two different continuous positive airway pressure systems for the successful extubation of extremely low birth weight infants. Pediatrics. 2003; 112:1031–1038.

8. Barrington KJ, Bull D, Finer NN. Randomized trial of nasal synchronized intermittent mandatory ventilation compared with continuous positive airway pressure after extubation of very low birth weight infants. Pediatrics. 2001; 107:638–641.

9. Davis PG, Henderson-Smart DJ. Nasal continuous positive airways pressure immediately after extubation for preventing morbidity in preterm infants. Cochrane Database Syst Rev. 2003;(2):CD000143.

10.Greenough A. Long-term pulmonary outcome in the preterm infant. Neonatology. 2008; 93: 324-7.

11.Chien YH, Tsao PN, Chou HC, Tang JR, Tsou KI. Rehospitalization of extremely-low-birth-weight infants in first 2 years of life. Early Hum Dev. 2002; 66: 33-40.

12. Lemyre B, Davis PG, De Paoli AG, Kirpalani H. Nasal intermittent positive pressure ventilation (NIPPV) versus nasal continuous positive airway pressure (NCPAP) for preterm neonates after extubation. Cochrane Database Syst Rev. 2017;(2):CD003212.

13. De Luca D, Dell'Orto V. Non-invasive high-frequency oscillatory ventilation in neonates: review of physiology, biology and clinical data. Arch Dis Child Fetal Neonatal Ed. 2016 Jun 28. pii: fetalneonatal-2016-310664. doi: 10.1136/archdischild-2016-310664.

14. Czernik C, Schmalisch G, Bührer C, Proquitté H. Weaning of neonates from mechanical ventilation by use of nasopharyngeal high frequency oscillatory ventilation: a preliminary study. J Matern Fetal Neonatal Med. 2012; 25: 374–378.

15. Mukerji A, Sarmiento K, Lee B, Hassall K, Shah V. Non-invasive high-frequency ventilation versus bi-phasic continuous positive airway pressure (BP-CPAP) following CPAP failure in infants <1250 g: a pilot randomized controlled trial. J Perinatol. 2017;37:49-53.

16.De Luca D. Noninvasive high-frequency ventilation and the errors from the past: designing simple trials neglecting complex respiratory physiology.J Perinatol. 2017;37:1065-1066.

17. Fisher HS, Bohlin K, Bührer C, Schmalisch G, Cremer M, Reiss I, Czernik C. Nasal high-frequency oscillation ventilation in neonates: a survey in five European Countries. Eur J Pediatr. 2015;174:465–71.

18. Zwarenstein M, Treweek S, Gagnier JJ, Altman DG, Tunis S, Haynes B, Oxman AD, Moher D; CONSORT group; Pragmatic Trials in Healthcare (Practihc) group. Improving the reporting of pragmatic trials: an extension of the CONSORT statement.
BMJ. 2008;337:a2390.

19. Restrepo RD, Hirst KR, WittnebelL, Wettstein R. AARC clinical practice guideline: transcutaneous monitoring of carbon dioxide and oxygen: 2012. Respir Care 2012;57:1955-1962.

20. Silverman WC, Anderson DH. Controlled clinical trial on effects of water mist on obstructive respiratory signs, death rate and necropsy findings among premature infants. Pediatrics. 1956; 17: 1-4.

21. Papile LA, Munsick-Bruno G, Schaefer A. Relationship of cerebral intraventricular hemorrhage and early childhood neurologic handicaps. J Pediatr. 1983;103:273-7.

22.Watkins AM, West CR, Cooke RW. Blood pressure and cerebral hemorrhage and ischaemia in very low birth weight infants. Early Hum Dev. 1989;19:103-10.

23. De Luca D, Romain O, Yousef N, Andriamanamirijaa D, Shankar-Aguilera S, Walls E, Sgaggero B, Aube N, Tissières P. Monitorages physiopathologiques en reanimation néonatale. J Ped Puericul. 2015; 28: 276-300. [Article in French]

24. Jobe AH, Bancalari E. Bronchopulmonary dysplasia. Am J Respir Crit Care Med. 2001;163:1723-9.

25. International Committee for the Classification of Retinopathy of Prematurity. The International Classification of Retinopathy of Prematurity revisited. Arch Ophthalmol. 2005;123:991-9.

26. Stoll BJ. Epidemiology of necrotizing enterocolitis. Clin Perinatol. 1994;21:205-18.

27. Fischer C, Bertelle V, Hohlfeld J, Forcada-Guex M, Stadelmann-Diaw C, Tolsa JF. Nasal trauma due to continuous positive airway pressure in neonates. Arch Dis Child Fetal Neonatal Ed. 2010;95:F447-51.

28. Stevens B, Johnston C, Petryshen P, Taddio A. Premature infant pain profile :developmend and initial validation. Clin J Pain. 1996;12:13-2

29. De Paoli AG, Davis PG, Faber B, Morley CJ. Devices and pressure sources for administration of nasal continuous positive airway pressure (NCPAP) in preterm neonates.
Cochrane Database Syst Rev. 2002; 3:CD002977

30. Roberts CT, Davis PG, Owen LS. Neonatal non-invasive respiratory support: synchronised NIPPV, non-synchronised NIPPV or bi-level CPAP: what is the evidence in 2013?
Neonatology. 2013;104:203-9.

31. Mukerji A, Belik J. Neonatal nasal intermittent positive pressure ventilation efficacy and lung pressure transmission.
J Perinatol. 2015;35:716–19.

32. Gerdes JS, Sivieri EM, Abbasi S. Factors influencing delivered mean airway pressure during nasal CPAP with the RAM cannula.
Pediatr Pulmonol. 2016;51:60–9.

33. De Jaegere A, van Veenendaal MB, Michiels A, van Kaam AH. Lung recruitment using oxygenation during openlunghigh-frequency ventilation in preterm infants.
Am J Respir Crit Care Med. 2006;174:639-45.

34. De Luca D, Piastra M, Pietrini D, Conti G. Effect of amplitude and inspiratory time in a bench model of non-invasive HFOV through nasal prongs. Pediatr Pulmonol. 2012;47:1012–18.

35. De Luca D, Carnielli VP, Conti G, Piastra M. Noninvasive high frequency oscillatory ventilation through nasal prongs: bench evaluation of efficacy and mechanics.
Intensive Care Med. 2010;36:2094–100.

36. Mukerji A, Finelli M, Belik J. Nasal high-frequency oscillation for lung carbon dioxide clearance in the newborn. Neonatology. 2013;103:161–5.

37. De Luca D, Costa R, Visconti F, Piastra M, Conti G. Oscillation transmission and volume delivery during face mask-delivered HFOV in infants: Bench and in vivo study. Pediatr Pulmonol. 2016;51:705-12.

38. Dell'Orto V, Centorrino R, Shankar-Aguilera S, Ben-Ammar R, Yousef N. De Luca D. Noninvasive high frequency oscillatory ventilation versus noninvasive intermittent positive pressure ventilation for preterm babies developing BPD: a pilot study. E-PAS 2018 in press (Abstract to the Pediatric Academic Societies meeting 2018).

39. Ramanathan R, Sekar KC, Rasmussen M, Bhatia J, Soll RF. Nasal intermittent positive pressure ventilation after surfactant treatment for respiratory distress syndrome in preterm infants <=30 weeks' gestation: a randomized, controlled trial. J Perinatol. 2012;32:336-43.

40. Faul F, Erdfelder E, Lang AG, Buchner A. G*Power 3: A flexible statistical power analysis program for the social, behavioral, and biomedical sciences. Behavior Research Methods. 2003;39:175-191.

41.Parry G, Tucker J, Tarnow-Mordi W; UK Neonatal Staffing Study Collaborative Group. CRIB II: an update of the clinical risk index for babies score. Lancet. 2003;24;361:1789-91.

42. Allison PD. Logistic regression using the SAS system. Cary (NC, USA): SAS Institute Ed; 1999.

43. Norusis M. SPSS 13.0 advanced statistical procedures companion. Upper Saddle-River (NJ, USA): Prentice Hall; 2004.

44. Shankar-Aguilera S, Taveira M, De Luca D. Neonatal ventilation trials need specific funding. Lancet Respir Med. 2014;2:867-869.

45.De Luca D, van Kaam AH, Tingay DG, Courtney SE, Danhaive O, Carnielli VP, Zimmermann LJ, Kneyber MCJ, Tissieres P, Brierley J, Conti G, Pillow JJ, Rimensberger PC. The Montreux definition of neonatal ARDS: biological and clinical background behind the description of a new entity. Lancet Respir Med. 2017;5:657-666.

46. De Luca D, Baroni S, Vento G, Piastra M, Pietrini D, Romitelli F, Capoluongo E, Romagnoli C, Conti G, Zecca E. Secretory phospholipase A2 and neonatal respiratory distress: pilot study on broncho-alveolar lavage. Intensive Care Med. 2008;34:1858-64.

47. Goldstein B, Giroir B, Randolph A; International Consensus Conference on Pediatric Sepsis.International pediatric sepsis consensus conference: definitions for sepsis and organ dysfunction in pediatrics. Pediatr Crit Care Med. 2005;6:2-8.

48. Piastra M, Yousef N, Brat R, Manzoni P, Mokhtari M, De Luca D. Lung ultrasound findings in meconium aspiration syndrome. Early Hum Dev. 2014;90 Suppl2:S41-3.

49. Contentin L, Ehrmann S, Giraudeau B. Heterogeneity in the definition of mechanical ventilation duration and ventilator-free days. Am J Respir Crit Care Med. 2014;15;189:998-1002.

INTENTIONALLY LEFT BLANK

INTENTIONALLY LEFT BLANK
