# Supplementary material for: Effectiveness of Nasal Continuous Positive Airway Pressure vs Nasal Intermittent Positive Pressure Ventilation vs Noninvasive High-Frequency Oscillatory Ventilation as Support After Extubation of Neonates Born Extremely Preterm or With More Severe Respiratory Failure: A Secondary Analysis of a Randomized Clinical Trial
Source: JAMA Netw Open. 2023 Jul 3;6(7):e2321644. doi: 10.1001/jamanetworkopen.2023.21644 (PMC10318479; doi:10.1001/jamanetworkopen.2023.21644)
Supplement: Supplement 2. — eFigure 1. CONSORT Flowchart of the Original NASONE Trial eTable 1. Basic Characteristics of Population Subgroups Complementary to the Subgroups of Interest (i.e.: Subgroups With More Mature Neonates and Those With Less Severe Respiratory Failure) eAppendix. P Values for Figures eTable 2. Reasons for Reintubation in the Subgroups eTable 3. Interaction Analyses for All Coprimary Outcomes eTable 4. Expected False-Positive Rates for All Coprimary Outcomes eFigure 2. Kaplan-Meier Analysis for Reintubations eFigure 3. Secondary Outcomes: Use of Postnatal Steroids (A), In-Hospital Mortality (B) and BPD/Mortality (C) eFigure 4. Secondary Outcomes: Airleaks (A), Number of Apneas/Week (B) and PIPP Score (C) eFigure 5. Secondary Outcomes: Weekly Weight Gain (A) and Severe Nasal Skin Injury (B) eFigure 6. Secondary Outcomes: IVH (A), NEC (B) and ROP (C) eFigure 7. Secondary Outcomes: Duration of Oxygen Supplementation (A) and of Study Intervention (B) [file jamanetwopen-e2321644-s002.pdf]

Supplemental Online Content

Zhu X, Li F, Shi Y, Feng Z, De Luca D; Nasal Oscillation Post-Extubation (NASONE) Study Group. Effectiveness of nasal continuous positive airway pressure vs nasal intermittent positive pressure ventilation vs noninvasive high-frequency oscillatory ventilation as support after extubation of neonates born extremely preterm or with more severe respiratory failure: a secondary analysis of a randomized clinical trial. *JAMA Netw Open*. 2023;6(7):e2321644. doi:10.1001/jamanetworkopen.2023.21644

eFigure 1. CONSORT Flowchart of the Original NASONE Trial
eTable 1. Basic Characteristics of Population Subgroups Complementary to the Subgroups of Interest (i.e.: Subgroups With More Mature Neonates and Those With Less Severe Respiratory Failure)
eAppendix. *P* Values for Figures
eTable 2. Reasons for Reintubation in the Subgroups
eTable 3. Interaction Analyses for All Coprimary Outcomes
eTable 4. Expected False-Positive Rates for All Coprimary Outcomes
eFigure 2. Kaplan-Meier Analysis for Reintubations
eFigure 3. Secondary Outcomes: Use of Postnatal Steroids (A), In-Hospital Mortality (B) and BPD/Mortality (C)
eFigure 4. Secondary Outcomes: Airleaks (A), Number of Apneas/Week (B) and PIPP Score (C)
eFigure 5. Secondary Outcomes: Weekly Weight Gain (A) and Severe Nasal Skin Injury (B)
eFigure 6. Secondary Outcomes: IVH (A), NEC (B) and ROP (C)
eFigure 7. Secondary Outcomes: Duration of Oxygen Supplementation (A) and of Study Intervention (B)

This supplemental material has been provided by the authors to give readers additional information about their work.

© 2023 Zhu X et al. *JAMA Network Open.*

eFigure 1. CONSORT Flowchart of the Original NASONE Trial. For the 80 neonates who met an exclusion criterion, the distribution was as follows: grade-IV IVH (n=21), major congenital anomalies/chromosomal abnormalities (n=9), upper respiratory tract abnormalities (n=11), need for surgery known before the first extubation (n=35), birth weight <600g (n=4). The allocated treatment was discontinued in 53 neonates because their parents/guardians withdrew their consent to participation in the trial.

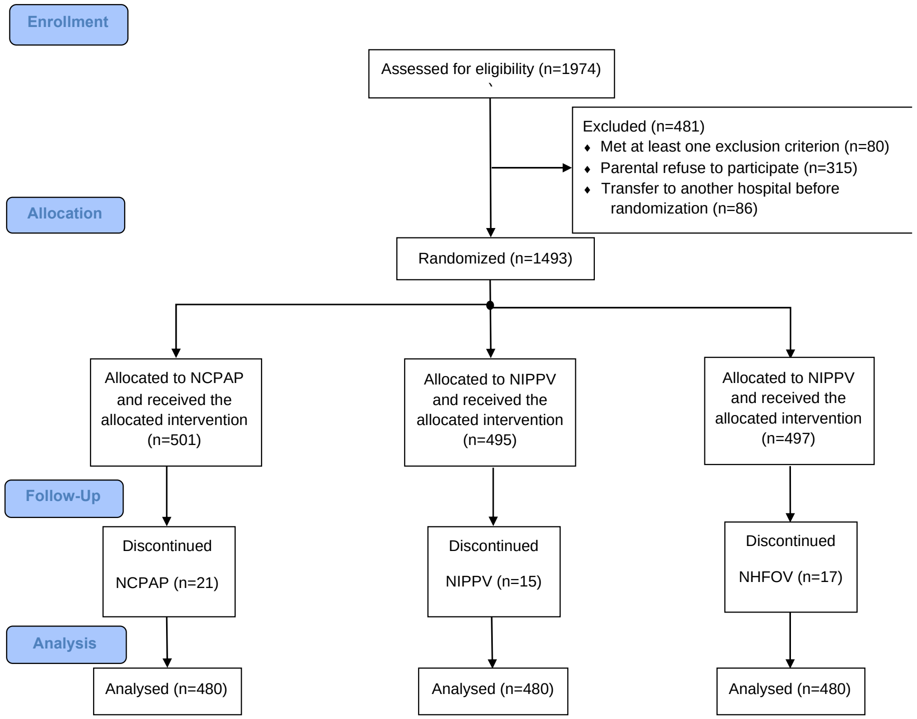

© 2023 Zhu X et al. *JAMA Network Open.*

eTable 1. Basic Characteristics of Population Subgroups Complementary to the Subgroups of Interest (i.e.: Subgroups With More Mature Neonates and Those With Less Severe Respiratory Failure). Data are expressed as mean (standard deviation) or number (%). Prenatal steroid is considered if complete (two 12 mg doses of betamethasone, 24 h apart from each other). Surfactant replacement was always performed by intubation-surfactant-extubation technique. CRIB-II, OI and pH are dimensionless variables. **Abbreviations**: CRIB-II: Clinical Risk Index for Babies-II score; FiO_2: inspired oxygen faction; NCPAP: nasal continuous positive airway pressure; NHFOV: noninvasive high-frequency oscillation ventilation; NIPPV: noninvasive positive pressure ventilation; OI: oxygenation index (OI=(FiO_2×Paw)×100/PaO_2); $PaCO_2$: partial pressure of carbon dioxide; PaO_2: partial oxygen pressure; Paw: mean airway pressure; SGA: small for gestational age.

	**>28 weeks' gestation**				**Ventilated < 1week from birth**				**CO_2 ≤ 50mmHg before or in the 24h after the extubation**			
	**NCPAP (N=346)**	**NIPPV (N=320)**	**NHFOV (N=319)**	***p***	**NCPAP (N=370)**	**NIPPV (N=349)**	**NHFOV (N=346)**	***p***	**NCPAP (N=357)**	**NIPPV (N=386)**	**NHFOV (N=390)**	***p***
Gestational age (weeks)	30.4 (1.1)	30.4 (1.1)	30.5 (1.1)	0.55	29.7 (1.6)	29.5 (1.8)	29.6 (1.8)	0.22	29.7 (1.6)	29.4 (1.8)	29.5 (1.8)	0.17
Birth weight (g)	1441 (298)	1482 (343)	1461 (330)	0.27	1382 (304)	1358 (353)	1369 (362)	0.64	1378 (318)	1340 (359)	1348 (353)	0.29
Male sex	191 (55.2%)	192 (60%)	198 (62%)	0.18	214 (57.8%)	213 (61%)	215 (62.1%)	0.47	203 (56.9%)	233 (60.4%)	241 (61.8%)	0.37
SGA neonates	36 (10.1%)	29 (9%)	21 (6.6%)	0.21	35 (9.5%)	34 (9.7%)	22 (6.4%)	0.21	34 (9.5%)	36 (9.3%)	26 (6.7%)	0.28
Twins	94 (27.2%)	97 (30.3%)	97 (30.4%)	0.57	98 (26.5%)	110 (31.5%)	103 (29.8%)	0.32	102 (28.6%)	126 (32.6%)	127 (32.6%)	0.40
Cesarean section	210 (60.7%)	198 (61.9%)	204 (63.9%)	0.68	202 (54.6%)	184 (52.7%)	180 (52%)	0.77	191 (53.5%)	211 (54.7%)	208 (53.3%)	0.92
Prenatal steroids	144 (41.6%)	161 (50.3%)	137 (42.9%)	0.06	161 (43.5%)	181 (51.9%)	169 (48.8%)	0.07	149 (41.7%)	194 (50.2%)	179 (45.9%)	0.06
CRIB-II score	4.1 (2.2)	3.9 (2.4)	4 (2.4)	0.58	4.7 (2.8)	4.9 (3)	5 (3.2)	0.34	4.9 (2.9)	5.1 (3.2)	5.3 (3.2)	0.24
5' Apgar score	9 [9-10]	9 [8-10]	9 [9-10]	0.87	9 [8-10]	9 [8-10]	9 [9-10]	0.78	9 [9-10]	9 [9-10]	9 [8-10]	0.67
Surfactant replacement	287 (82.9%)	266 (83.1%)	259 (81.2%)	0.77	307 (83%)	296 (84.8%)	283 (81.2%)	0.56	299 (83.8%)	330 (85.5%)	322 (82.6%)	0.53
Early onset sepsis	10 (2.9%)	6 (1.9%)	8 (2.5%)	0.69	8 (2.2%)	8 (2.3%)	9 (2.6%)	0.92	7 (2%)	7 (1.8%)	7 (1.8%)	0.98

© 2023 Zhu X et al. *JAMA Network Open.*

eAppendix. *P* Values for Figures

For Figure 1A in the text, for neonates of gestational age 28 or fewer weeks, *P* = .007 for noninvasive positive pressure ventilation (NIPPV) vs nasal continuous positive airway pressure (NCPAP), and *P* < .001 for noninvasive high-frequency oscillation ventilation (NHFOV) vs NCPAP. For neonates invasively ventilated for at least 1 week, *P* < .001 for NIPPV vs NCPAP and NHFOV vs NCPAP. For neonates with CO_2 greater than 50 mm Hg before or in the 24 hours after extubation, *P* = .003 for NIPPV vs NCPAP, and *P* < .001 for NHFOV vs NCPAP. For Figure 1B in the text, for neonates of gestational age 28 or fewer weeks, *P* < .001 for NIPPV vs NCPAP and NHFOV vs NCPAP. For neonates invasively ventilated for at least 1 week, *P* < .001 for NIPPV vs NCPAP and NHFOV vs NCPAP. For neonates with CO_2 greater than 50 mm Hg before or in the 24 hours after extubation, *P* < .001 for NIPPV vs NCPAP, and *P* = .01 for NHFOV vs NCPAP.

For Figure 2A in the text, for the mean difference in IMV duration in neonates of gestational age up to 28 weeks, *P* = .03 for noninvasive positive pressure ventilation (NIPPV) vs nasal continuous positive airway pressure (NCPAP), and *P* = .008 for noninvasive high-frequency oscillation ventilation (NHFOV) vs NCPAP. For the mean difference in IMV duration in neonates invasively ventilated for at least 1 week, *P* < .001 for NIPPV vs NCPAP and NHFOV vs NCPAP, and *P* = .03 for NIPPV vs NHFOV. For the mean difference in IMV duration in neonates with CO_2 greater than 50 mm Hg before or in the 24 hours after extubation, *P* = .04 for NHFOV vs NCPAP. For Figure 2B in the text, for the mean difference in ventilator-free days for neonates with CO_2 greater than 50 mm Hg before or in the 24 hours after extubation, *P* = .04 for NIPPV vs NCPAP.

For Figure 3A in the text, for the difference in risk of BPD in neonates of gestational age 28 or fewer weeks, *P* = .01 for noninvasive high-frequency oscillation ventilation (NHFOV) vs nasal continuous positive airway pressure (NCPAP). For Figure 3B in the text, for the difference in risk of moderate-to-severe BPD in neonates of gestational age 28 or fewer weeks, *P* = .04 for NHFOV vs NCPAP. For the difference in risk of moderate-to-severe BPD in neonates in neonates invasively ventilated for at least 1 week, *P* = .02 for NHFOV vs NCPAP. For the difference in risk of moderate-to-severe BPD in neonates with CO_2 greater than 50 mm Hg before or in the 24 hours after extubation, *P* = .03 for NHFOV vs NCPAP.

For Figure 4A in the text, for the difference in oxygenation index for neonates of gestational age 28 or fewer weeks, *P* = .001 for noninvasive high-frequency oscillation ventilation (NHFOV) vs nasal continuous positive airway pressure (NCPAP), and *P* = .05 for noninvasive positive pressure ventilation (NIPPV) vs NHFOV. For the difference in oxygenation index for neonates invasively ventilated for at least 1 week, *P* = .04 for NHFOV vs NCPAP, and *P* = .007 for NIPPV vs NHFOV. For the difference in oxygenation index for neonates with CO_2 greater than 50 mm Hg before or in the 24 hours after extubation, *P* = .005 for NHFOV vs NCPAP, and *P* = .04 for NIPPV vs NHFOV. For Figure 4B in the text, for the difference in CO_2 for neonates of gestational age 28 or fewer weeks, *P* = .02 for NHFOV vs NCPAP. For the difference in CO_2 for neonates invasively ventilated for at least 1 week, *P* = .04 for NIPPV vs NCPAP, and *P* = .02 for NHFOV vs NCPAP.

© 2023 Zhu X et al. *JAMA Network Open.*

eTable 2. Reasons for Reintubation in the Subgroups. Raw data are expressed as number (%); differences between study arms are expressed as risk difference (95% CI) for the re-intubations. The frequency of refractory hypoxemia in the subgroup of interest is significantly different (neonates ≤28^{+6} weeks' gestation: overall *p*=0.006; neonates ventilated ≥1week from birth: overall *p*=0.0003; neonates with CO_2 > 50mmHg before or in the 24h after the extubation: overall *p*=0.001). **Abbreviations:** NCPAP: nasal continuous positive airway pressure; NHFOV: non-invasive high-frequency oscillation ventilation; NIPPV: noninvasive positive pressure ventilation.

**Neonates ≤28^{+6} weeks' gestation**									
	**NCPAP (N=134)**	**NIPPV (N=160)**	**NHFOV (N=161)**	**NCPAP - NIPPV**		**NCPAP - NHFOV**		**NIPPV - NHFOV**	
				**Difference**	***Post-hoc p***	**Difference**	***Post-hoc p***	**Difference**	***Post-hoc p***
Severe respiratory acidosis	7(5.2%)	4(2.5%)	3(1.8%)	2.7(-1.8;8.1)	0.24	3.3(-1.0;8.7)	0.19	0.6(-3.1;4.6)	0.72
Refractory hypoxemia	36(26.9%)	28(17.5%)	22(13.7%)	9.4(-0.1;18.9)	**0.05**	13.2(4.0;22.4)	**0.005**	3.8(-4.2;11.8)	0.34
Severe apneas	11(8.2%)	9(5.6%)	7(4.3%)	2.6(-3.3;9.0)	0.38	3.8(-1.8;10.2)	0.17	1.3(-3.8;6.5)	0.60
Pulmonary hemorrhage	1(0.7%)	0	0	0	--	0	--	0	--
Hemodynamic instability	1(0.7%)	3(1.9)	3(1.8%)	-1.1(-2.4;4.7)	0.63	-1.1(-2.5;4.6)	0.63	0(-2.8;2.9)	>0.99
Cardio-respiratory arrest	1(0.7%)	0	0	0	--	0	--	0	--
**Neonates >28 weeks' gestation**									
	**NCPAP (N=346)**	**NIPPV (N=320)**	**NHFOV (N=319)**	**NCPAP - NIPPV**		**NCPAP - HFOV**		**NIPPV - NHFOV**	
				**Difference**	***Post-hoc p***	**Difference**	***Post-hoc p***	**Difference**	***Post-hoc p***
Severe respiratory acidosis	14(4%)	4(1.2%)	3(0.9%)	2.8(0.3;5.5)	**0.03**	3.1(0.7;5.8)	**0.01**	0.3(-1.6;2.3)	>0.99
Refractory hypoxemia	41(11.8%)	25(7.8%)	16(5%)	4.0(-0.5;8.6)	0.08	6.8(2.6;11.1)	**0.002**	2.8(-1.1;6.7)	0.15
Severe apneas	5(1.4%)	3(0.9%)	2(0.6%)	0.5(-1.4;2.5)	0.73	0.8(-1.0;2.7)	0.45	0.3(-1.4;2.1)	>0.99
Pulmonary hemorrhage	1(0.3%)	1(0.3%)	1(0.3%)	0(-1.3;1.5)	>0.99	0(-1.3;1.5)	>0.99	0(-1.3;1.5)	>0.99
Hemodynamic instability	4(1.1%)	4(1.2%)	4(1.2%)	-0.1(-1.8;2.1)	>0.99	-0.1(-1.8;2.1)	>0.99	0(-2.0;2.0)	>0.99
Cardio-respiratory arrest	1(0.3%)	3(0.9%)	2(0.6%)	-0.6(-0.8;2.4)	0.35	-0.3(-1.4;2.1)	0.61	0.3(-1.4;2.1)	>0.99
**Neonates ventilated ≥ 1week from birth**									
	**NCPAP (N=110)**	**NIPPV (N=131)**	**NHFOV (N=134)**	**NCPAP - NIPPV**		**NCPAP - HFOV**		**NIPPV - NHFOV**	
				**Difference**	***Post-hoc p***	**Difference**	***Post-hoc p***	**Difference**	***Post-hoc p***
Severe respiratory acidosis	5(4.5%)	2(1.5%)	1(0.7%)	3.0(-1.6;8.8)	0.25	3.8(-0.4;9.5)	0.09	0.8(-2.7;4.7)	0.62
Refractory hypoxemia	31(28.2%)	16(12.2%)	14(10.4%)	16.0(5.8;26.1)	**0.002**	17.7(7.9;27.6)	**<0.001**	1.8(-6.0;9.6)	0.65
Severe apneas	8(7.3%)	6(4.6%)	3(2.2%)	2.7(-3.5;9.6)	0.37	5.0(-0.4;11.6)	0.07	2.3(-2.5;7.6)	0.33
Pulmonary hemorrhage	1(0.9%)	0	0	0	--	0	--	0	--
Hemodynamic instability	1(0.9%)	1(0.7%)	0	>0.99	0.1(-3.4;4.2)	0	--	0	--
Cardio-respiratory arrest	0	0	0	0	--	0	--	0	--
**Ventilated < 1week from birth**									
	**NCPAP (N=370)**	**NIPPV (N=349)**	**NHFOV (N=346)**	**NCPAP - NIPPV**		**NCPAP - NHFOV**		**NIPPV - NHFOV**	
				**Difference**	***Post-hoc p***	**Difference**	***Post-hoc p***	**Difference**	***Post-hoc p***
Severe respiratory acidosis	16(4.3%)	6(1.7%)	5(1.4%)	2.6(0.0;5.3)	**0.04**	2.9(0.4;5.6)	**0.02**	0.3(-1.8;2.4)	0.99
Refractory hypoxemia	46(12.4%)	37(10.6%)	24(6.9%)	1.8(-2.3;6.5)	0.53	5.5(1.1;9.8)	**0.01**	3.7(-0.6;7.9)	0.07

© 2023 Zhu X et al. *JAMA Network Open.*

Severe apneas	8(2.1%)	6(1.7%)	6(1.7%)	0.4(-1.8;2.7)	0.67	0.4(-1.8;2.7)	0.68	0(-2.2;2.2)	>0.99
Pulmonary hemorrhage	1(0.3%)	1(0.3%)	1(0.3%)	0(-1.3;1.3)	>0.99	0(-1.3;1.3)	>0.99	0(-1.3;1.3)	>0.99
Hemodynamic instability	4(1.1%)	6(1.7%)	7(2%)	-0.6(-1.3;2.7)	0.54	-0.9(-1.0;3.1)	0.37	-0.3(-1.9;2.6)	0.77
Cardio-respiratory arrest	2(0.5%)	3(0.8%)	2(0.6%)	-0.3(-1.2;2.0)	0.67	0(-1.4;1.6)(	>0.99	0.3(-1.3;2.0)	>0.99
**Neonates with CO_2 > 50mmHg before or in the 24h after the extubation**									
	**NCPAP (N=123)**	**NIPPV (N=94)**	**NHFOV (N=90)**	**NCPAP - NIPPV**		**NCPAP - NHFOV**		**NIPPV - NHFOV**	
				**Difference**	***Post-hoc p***	**Difference**	***Post-hoc p***	**Difference**	***Post-hoc p***
Severe respiratory acidosis	6(4.9%)	2(2.1%)	2(2.2%)	2.7(-3.1;8.3)	0.47	2.6(-3.4;8.2)	0.47	-0.1(-5.4;5.8)	>0.99
Refractory hypoxemia	45(36.6%)	17(18.1%)	16(17.8%)	18.5(6.5;29.4)	**0.003**	18.8(6.6;29.7)	**0.003**	0.3(-10.9;11.4)	>0.99
Severe apneas	11(8.9%)	7(7.4%)	4(4.4%)	1.5(-6.6;8.9)	0.69	4.5(-3.0;11.1)	0.28	3.0(-4.5;10.6)	0.54
Pulmonary hemorrhage	0	0	0	0	--	0	--	0	--
Hemodynamic instability	2(1.6%)	4(4.2%)	3(3.3%)	-2.6(-2.2;8.9)	0.41	-1.7(-2.9;7.8)	0.65	0.9(-5.6;7.5)	>0.99
Cardio-respiratory arrest	0	0	0	0	--	0	--	0	--
**Neonates with CO_2 ≤ 50mmHg before or in the 24h after the extubation**									
	**NCPAP (N=357)**	**NIPPV (N=386)**	**NHFOV (N=390)**	**NCPAP - NIPPV**		**NCPAP - NHFOV**		**NIPPV - NHFOV**	
				**Difference**	***Post-hoc p***	**Difference**	***Post-hoc p***	**Difference**	***Post-hoc p***
Severe respiratory acidosis	15(4.2%)	6(1.5%)	4(1%)	2.6(0.2;5.4)	**0.03**	3.2(0.9;5.8)	**0.009**	0.5(-1.2;2.4)	0.54
Refractory hypoxemia	32(8.9%)	36(9.3%)	22(5.6%)	-0.3(-3.9;4.5)	0.78	3.3(-0.4;7.2)	0.10	3.7(0;7.5)	0.05
Severe apneas	5(1.4%)	5(1.3%)	5(1.3%)	0.1(-1.8;2.1)	>0.99	0.1(-1.7;2.1)	>0.99	0(-1.8;1.9)	>0.99
Pulmonary hemorrhage	2(0.5%)	1(0.2%)	1(0.2%)	0.3(-0.9;1.8)	0.61	0.3(-0.9;1.8)	0.61	0(-1.2;1.2)	>0.99
Hemodynamic instability	3(0.8%)	3(0.8%)	4(1%)	0(-1.5;1.7)	>0.99	-0.2(-1.5;1.8)	>0.99	-0.2(-1.3;1.9)	>0.99
Cardio-respiratory arrest	2(0.5%)	3(0.8%)	2(0.5%)	-0.2(-1.3;1.7)	>0.99	0(-1.3;1.5)	>0.99	0.2(-1.2;1.8)	>0.99

© 2023 Zhu X et al. *JAMA Network Open.*

eTable 3. Interaction Analyses for All Coprimary Outcomes. Data are expressed as hazard ratio (for reintubation and early reintubations) or β coefficient (for duration of invasive ventilation and ventilator-free days) relative to the interaction term between the study intervention and the variable used to define subgroups, per each regression model. **Abbreviations:** CI: confidence interval; HR: hazard ratio; IMV: invasive mechanical ventilation; VFD: ventilator-free days.

	**HR or β**	**95%CI**	***p***
**Neonates ≤28^{+6} weeks' gestation**			
Duration of IMV	0.09	-1.1; 1.2	0.945
Reintubations	1.007	0.82; 1.24	0.950
Early reintubations	1.001	0.77; 1.30	0.995
VFD	4.63	2.7; 10.2	**<0.001**
**Neonates ventilated ≥ 1week from birth**			
Duration of IMV	1.6	0.04; 0.9	**0.032**
Reintubations	0.959	0.83; 1.1	0.569
Early reintubations	1.05	0.9; 1.23	0.529
VFD	0.64	-0.79;2	0.395
**Neonates with CO_2 > 50mmHg**			
Duration of IMV	-0.24	-0.1: 0.04	0.367
Reintubations	1.005	0.98; 1.023	0.571
Early reintubations	1.01	0.99; 1.029	0.317
VFD	-0.42	-0.39;0.05	0.126

© 2023 Zhu X et al. *JAMA Network Open.*

eTable 4. Expected False-Positive Rates for All Coprimary Outcomes. **Abbreviations:** NCPAP: nasal continuous positive airway pressure; NHFOV: non-invasive high-frequency oscillation ventilation; NIPPV: noninvasive positive pressure ventilation; VFD: ventilator-free days.

**Reintubations**		**Duration IMV**	
**Neonates $\le 28^{+6}$ weeks' gestation**		**Neonates $\le 28^{+6}$ weeks' gestation**	
NCPAP vs NIPPV	0.018	NCPAP vs NIPPV	0.02
NCPAP vs NHFOV	0.007	NCPAP vs NHFOV	0.01
NIPPV vs NHFOV	0.04	NIPPV vs NHFOV	0.049
**Neonates ventilated ≥ 1week from birth**		**Neonates ventilated ≥ 1week from birth**	
NCPAP vs NIPPV	0.008	NCPAP vs NIPPV	0.008
NCPAP vs NHFOV	0.003	NCPAP vs NHFOV	0.03
NIPPV vs NHFOV	0.04	NIPPV vs NHFOV	0.03
**Neonates with CO_2 > 50mmHg**		**Neonates with CO_2 > 50mmHg**	
NCPAP vs NIPPV	0.014	NCPAP vs NIPPV	0.04
NCPAP vs NHFOV	0.004	NCPAP vs NHFOV	0.04
NIPPV vs NHFOV	0.04	NIPPV vs NHFOV	0.04
**Early reintubations**		**VFD**	
**Neonates $\le 28^{+6}$ weeks' gestation**		**Neonates $\le 28^{+6}$ weeks' gestation**	
NCPAP vs NIPPV	0.008	NCPAP vs NIPPV	0.03
NCPAP vs NHFOV	0.005	NCPAP vs NHFOV	0.049
NIPPV vs NHFOV	0.04	NIPPV vs NHFOV	0.04
**Neonates ventilated ≥ 1week from birth**		**Neonates ventilated ≥ 1week from birth**	
NCPAP vs NIPPV	0.007	NCPAP vs NIPPV	0.049
NCPAP vs NHFOV	0.006	NCPAP vs NHFOV	0.04
NIPPV vs NHFOV	0.049	NIPPV vs NHFOV	0.038
**Neonates with CO_2 > 50mmHg**		**Neonates with CO_2 > 50mmHg**	
NCPAP vs NIPPV	0.008	NCPAP vs NIPPV	0.02
NCPAP vs NHFOV	0.013	NCPAP vs NHFOV	0.04
NIPPV vs NHFOV	0.04	NIPPV vs NHFOV	0.04

© 2023 Zhu X et al. *JAMA Network Open.*

eFigure 2. Kaplan-Meier Analysis for Reintubations. Panel A, B and C show subgroup analysis for neonates ≤28 weeks' gestation, invasively ventilated for > 1 week and in those with CO_2 > 50mmHg, respectively. Blue, violet and turquoise lines depict NCPAP, NIPPV and NHFOV arms, respectively. Curves are different at Logrank test (*p*<0.001). Open circles represent censored cases. **Abbreviations**: NCPAP: nasal continuous positive airway pressure; NHFOV: non-invasive high-frequency oscillation ventilation; NIPPV: noninvasive positive pressure ventilation.

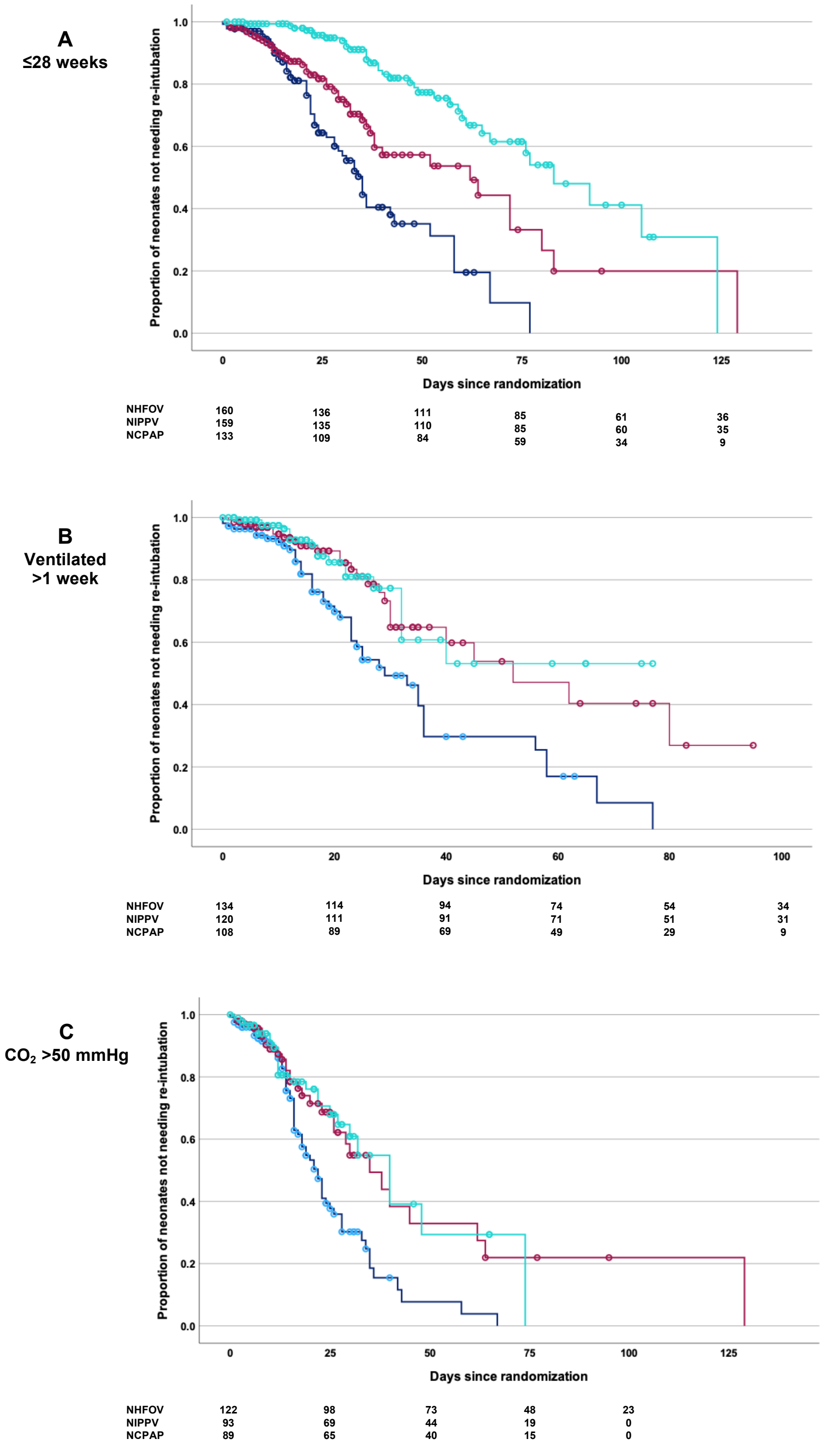

© 2023 Zhu X et al. *JAMA Network Open.*

eFigure 3. Secondary Outcomes: Use of Postnatal Steroids (A), In-Hospital Mortality (B) and BPD/Mortality (C). Subgroups of interest are highlighted with a grey background, alternate subgroups have no background. Data are shown as risk difference and 95% confidence interval and illustrated as Forrest plots per each subgroup. Squares and lines indicate the mean differences and their 95% confidence interval, respectively. Square size is proportional to the subgroup numerosity. Blue and green lines indicate comparisons significantly in favor of NIPPV and NHFOV, respectively (*p*-values for significant comparisons in subgroups of interest are as follows, **Panel C**: difference in risk of BPD/mortality composite endpoint in neonates with a gestational age ≤28 weeks: NHFOV vs NCPAP *p*=0.03). **Abbreviations**: BPD: bronchopulmonary dysplasia; CI: confidence interval; NCPAP: nasal continuous positive airway pressure; NHFOV: non-invasive high-frequency oscillation ventilation; NIPPV: noninvasive positive pressure ventilation.

A

Comparisons	Risk Difference (95%CI)	Steroids -treated neonates	Steroids -treated neonates
NCPAP vs NIPPV (<=28W)	0.103 (−0.003, 0.209)	49/134	42/160
NCPAP vs NHFOV (<=28W)	0.092 (−0.014, 0.199)	49/134	44/161
NIPPV vs NHFOV (<=28W)	−0.011 (−0.108, 0.086)	42/160	44/161
NCPAP vs NIPPV (>28W)	0.032 (−0.018, 0.082)	49/346	35/320
NCPAP vs NHFOV (>28W)	0.082 (0.037, 0.127)	49/346	19/319
NIPPV vs NHFOV (>28W)	0.050 (0.007, 0.093)	35/320	19/319
NCPAP vs NIPPV (Ventilated >=1W)	0.039 (−0.079, 0.157)	37/110	39/131
NCPAP vs NHFOV (Ventilated >=1W)	0.135 (0.023, 0.246)	37/110	27/134
NIPPV vs NHFOV (Ventilated >=1W)	0.096 (−0.007, 0.200)	39/131	27/134
NCPAP vs NIPPV (Ventilated <1W)	0.056 (0.006, 0.106)	61/370	38/349
NCPAP vs NHFOV (Ventilated <1W)	0.061 (0.011, 0.110)	61/370	36/346
NIPPV vs NHFOV (Ventilated <1W)	0.005 (−0.041, 0.051)	38/349	36/346
NCPAP vs NIPPV (CO2 >50 mmHg)	0.072 (−0.047, 0.192)	39/123	23/94
NCPAP vs NHFOV (CO2 >50 mmHg)	0.150 (0.038, 0.263)	39/123	15/90
NIPPV vs NHFOV (CO2 >50 mmHg)	0.078 (−0.038, 0.194)	23/94	15/90
NCPAP vs NIPPV (CO2 <=50 mmHg)	0.025 (−0.026, 0.077)	59/357	54/386
NCPAP vs NHFOV (CO2 <=50 mmHg)	0.042 (−0.008, 0.093)	59/357	48/390
NIPPV vs NHFOV (CO2 <=50 mmHg)	0.017 (−0.031, 0.064)	54/386	48/390

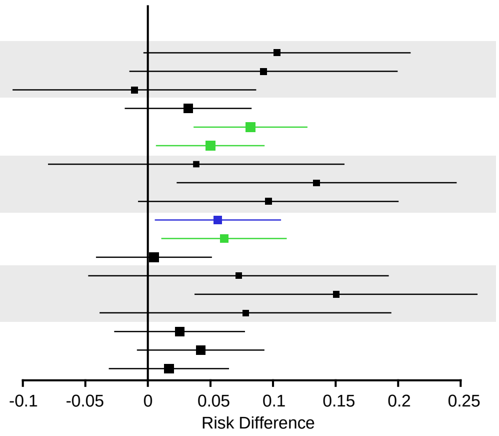

B

Comparisons	Risk Difference (95%CI)	Deaths	Deaths
NCPAP vs NIPPV (<=28W)	0.002 (−0.024, 0.029)	2/134	2/160
NCPAP vs NHFOV (<=28W)	−0.016 (−0.050, 0.018)	2/134	5/161
NIPPV vs NHFOV (<=28W)	−0.019 (−0.050, 0.013)	2/160	5/161
NCPAP vs NIPPV (>28W)	0.002 (−0.011, 0.015)	3/346	2/320
NCPAP vs NHFOV (>28W)	−0.001 (−0.015, 0.014)	3/346	3/319
NIPPV vs NHFOV (>28W)	−0.003 (−0.017, 0.011)	2/320	3/319
NCPAP vs NIPPV (Ventilated >=1W)	0.011 (−0.019, 0.040)	2/110	1/131
NCPAP vs NHFOV (Ventilated >=1W)	−0.012 (−0.050, 0.026)	2/110	4/134
NIPPV vs NHFOV (Ventilated >=1W)	−0.022 (−0.055, 0.010)	1/131	4/134
NCPAP vs NIPPV (Ventilated <1W)	−0.000 (−0.014, 0.013)	3/370	3/349
NCPAP vs NHFOV (Ventilated <1W)	−0.003 (−0.018, 0.011)	3/370	4/346
NIPPV vs NHFOV (Ventilated <1W)	−0.003 (−0.018, 0.012)	3/349	4/346
NCPAP vs NIPPV (CO2 >50 mmHg)	−0.016 (−0.058, 0.026)	2/123	3/94
NCPAP vs NHFOV (CO2 >50 mmHg)	−0.017 (−0.060, 0.026)	2/123	3/90
NIPPV vs NHFOV (CO2 >50 mmHg)	−0.001 (−0.053, 0.050)	3/94	3/90
NCPAP vs NIPPV (CO2 <=50 mmHg)	0.006 (−0.005, 0.017)	3/357	1/386
NCPAP vs NHFOV (CO2 <=50 mmHg)	−0.004 (−0.019, 0.010)	3/357	5/390
NIPPV vs NHFOV (CO2 <=50 mmHg)	−0.010 (−0.022, 0.002)	1/386	5/390

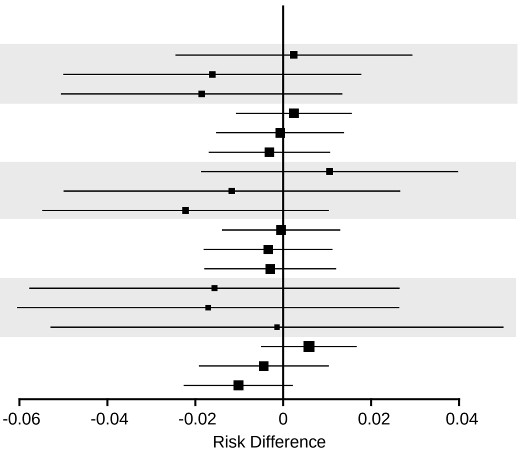

C

Comparisons	Risk Difference (95%CI)	BPD/ Deaths	BPD/ Deaths
NCPAP vs NIPPV (<=28W)	0.091 (−0.012, 0.195)	101/134	106/160
NCPAP vs NHFOV (<=28W)	0.120 (0.016, 0.224)	101/134	102/161
NIPPV vs NHFOV (<=28W)	0.029 (−0.075, 0.133)	106/160	102/161
NCPAP vs NIPPV (>28W)	0.004 (−0.062, 0.070)	88/346	80/320
NCPAP vs NHFOV (>28W)	0.038 (−0.026, 0.102)	88/346	69/319
NIPPV vs NHFOV (>28W)	0.034 (−0.032, 0.099)	80/320	69/319
NCPAP vs NIPPV (Ventilated >=1W)	0.040 (−0.086, 0.165)	64/110	71/131
NCPAP vs NHFOV (Ventilated >=1W)	0.097 (−0.028, 0.222)	64/110	65/134
NIPPV vs NHFOV (Ventilated >=1W)	0.057 (−0.063, 0.177)	71/131	65/134
NCPAP vs NIPPV (Ventilated <1W)	0.008 (−0.061, 0.077)	125/370	115/349
NCPAP vs NHFOV (Ventilated <1W)	0.031 (−0.037, 0.100)	125/370	106/346
NIPPV vs NHFOV (Ventilated <1W)	0.023 (−0.046, 0.092)	115/349	106/346
NCPAP vs NIPPV (CO2 >50 mmHg)	0.015 (−0.120, 0.149)	62/123	46/94
NCPAP vs NHFOV (CO2 >50 mmHg)	0.004 (−0.132, 0.140)	62/123	45/90
NIPPV vs NHFOV (CO2 >50 mmHg)	−0.011 (−0.155, 0.134)	46/94	45/90
NCPAP vs NIPPV (CO2 <=50 mmHg)	−0.007 (−0.076, 0.062)	127/357	140/386
NCPAP vs NHFOV (CO2 <=50 mmHg)	0.033 (−0.035, 0.101)	127/357	126/390
NIPPV vs NHFOV (CO2 <=50 mmHg)	0.040 (−0.027, 0.106)	140/386	126/390

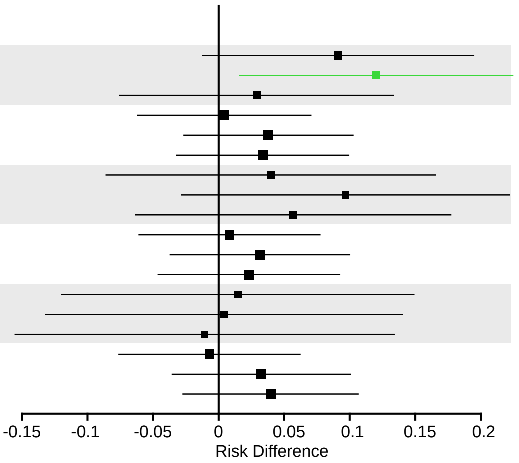

© 2023 Zhu X et al. *JAMA Network Open.*

eFigure 4. Secondary Outcomes: Airleaks (A), Number of Apneas/Week (B) and PIPP Score (C). Subgroups of interest are highlighted with a grey background, alternate subgroups have no background. PIPP is a dimensionless variable. Data are shown as risk difference and 95% confidence interval (for airleaks) and as mean difference and 95% confidence interval (for the number of apneas/week and the PIPP score). Data are illustrated as Forrest plots per each subgroup. Squares and lines indicate the mean differences and their 95% confidence interval, respectively. Square size is proportional to the subgroup numerosity. Blue, green and red lines indicate comparisons significantly in favor of NIPPV, NHFOV and NCPAP, respectively (*p*-values for significant comparisons in subgroups of interest are as follows, **Panel B**: difference in mean number of apneas/week in neonates invasively ventilated for at least 1 week: NIPPV *vs* NCPAP *p*=0.03, NHFOV vs NCPAP *p*=0.03; difference in mean number of apneas/week in neonates with CO_2 >50 mmHg before or in the 24h after the extubation: NIPPV *vs* NCPAP *p*=0.03, NHFOV vs NCPAP *p*=0.03). **Abbreviations**: CI: confidence interval; NCPAP: nasal continuous positive airway pressure; NHFOV: non-invasive high-frequency oscillation ventilation; NIPPV: noninvasive positive pressure ventilation; PIPP: premature infant pain profile.

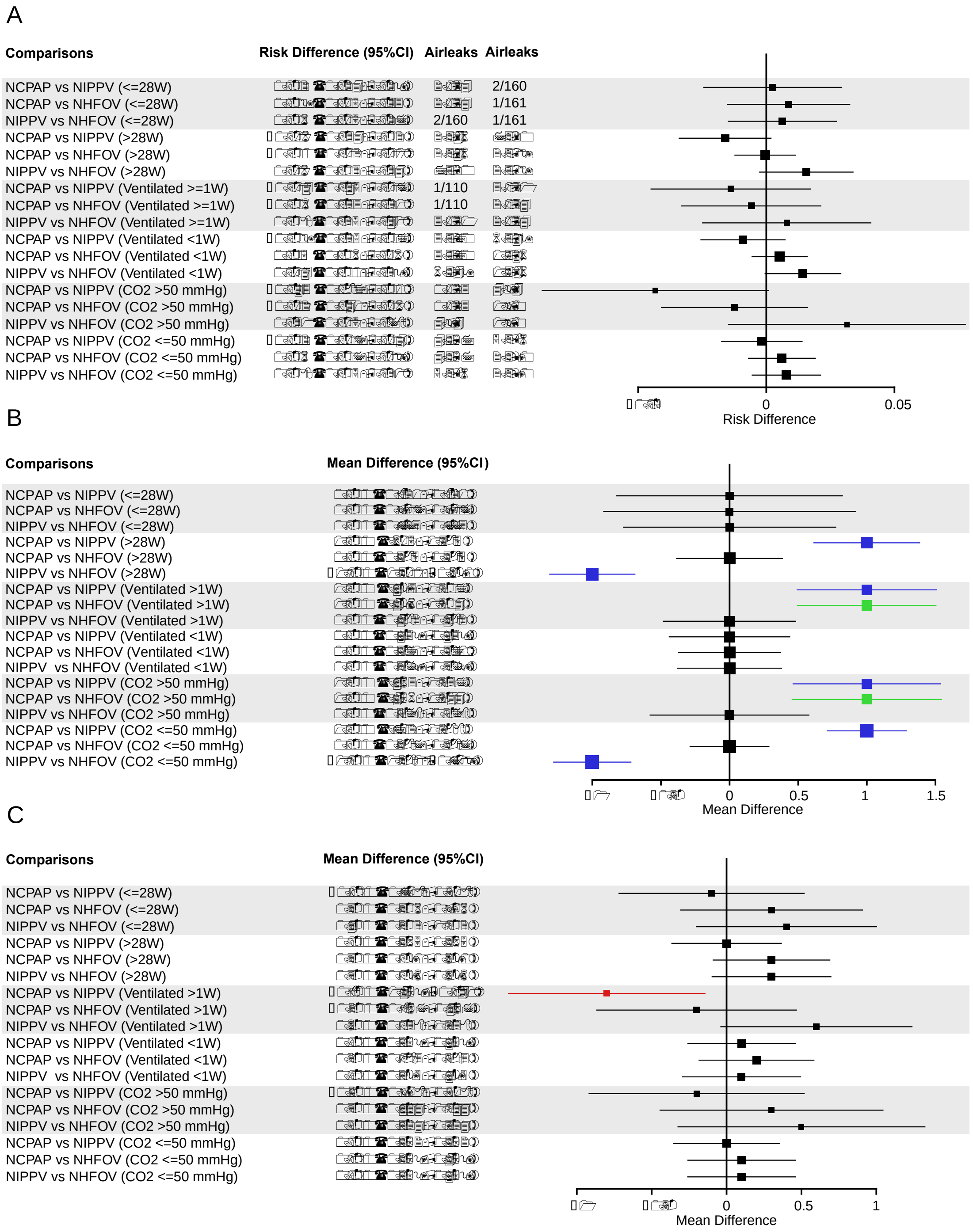

© 2023 Zhu X et al. *JAMA Network Open.*

eFigure 5. Secondary Outcomes: Weekly Weight Gain (A) and Severe Nasal Skin Injury (B). Subgroups of interest are highlighted with a grey background, alternate subgroups have no background. Weekly weight gain is expressed in grams; severe nasal skin injury was diagnosed if classified as grade III-IV using a dedicated dimensionless score; more details in the trial protocol). Data are shown as mean difference and 95% confidence interval (for weekly weight gain) and as risk difference and 95% confidence interval (for severe nasal skin injury); Data are illustrated as Forrest plots per each subgroup. Squares and lines indicate the mean differences and their 95% confidence interval, respectively. Square size is proportional to the subgroup numerosity. Blue and red lines indicate comparisons significantly in favor of NIPPV and NCPAP, respectively. **Abbreviations**: CI: confidence interval; NCPAP: nasal continuous positive airway pressure; NHFOV: non-invasive high-frequency oscillation ventilation; NIPPV: noninvasive positive pressure ventilation.

A

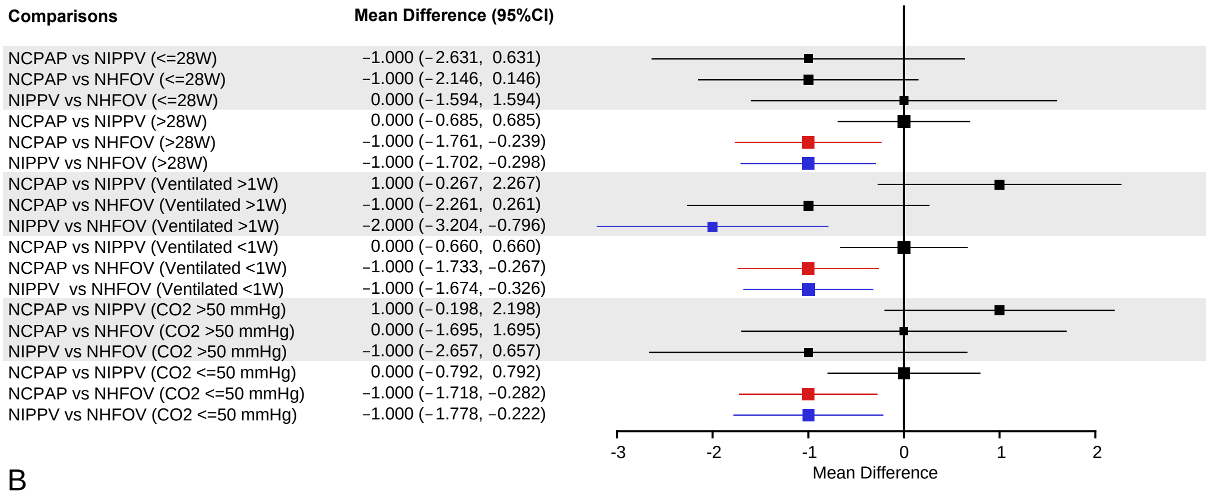

B

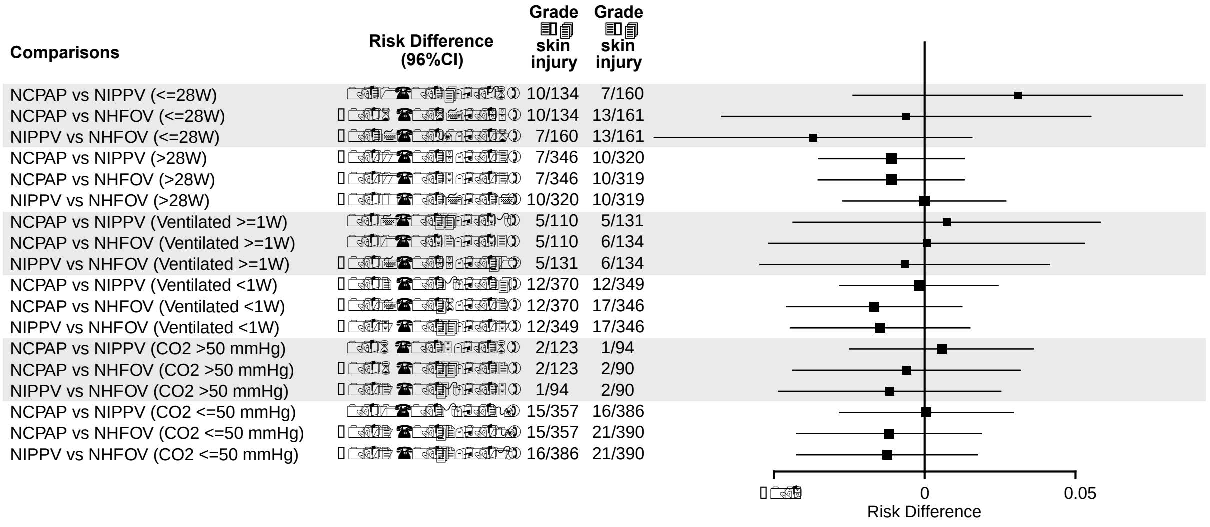

© 2023 Zhu X et al. *JAMA Network Open.*

eFigure 6. Secondary Outcomes: IVH (A), NEC (B) and ROP (C). Subgroups of interest are highlighted with a grey background, alternate subgroups have no background. IVH were considered if >2nd grade, NEC were considered if ≥2nd stage and ROP were considered if >2nd stage (more details in the trial protocol). Data are shown as risk difference and 95% confidence interval. Data are illustrated as Forrest plots per each subgroup. Squares and lines indicate the mean differences and their 95% confidence interval, respectively. Square size is proportional to the subgroup numerosity. Blue and green lines indicate comparisons significantly in favor of NIPPV and NHFOV, respectively. **Abbreviations**: CI: confidence interval; IVH: intra-ventricular hemorrhage; NEC: necrotizing entero-colitis; NCPAP: nasal continuous positive airway pressure; NHFOV: non-invasive high-frequency oscillation ventilation; NIPPV: noninvasive positive pressure ventilation; ROP: retinopathy of prematurity.

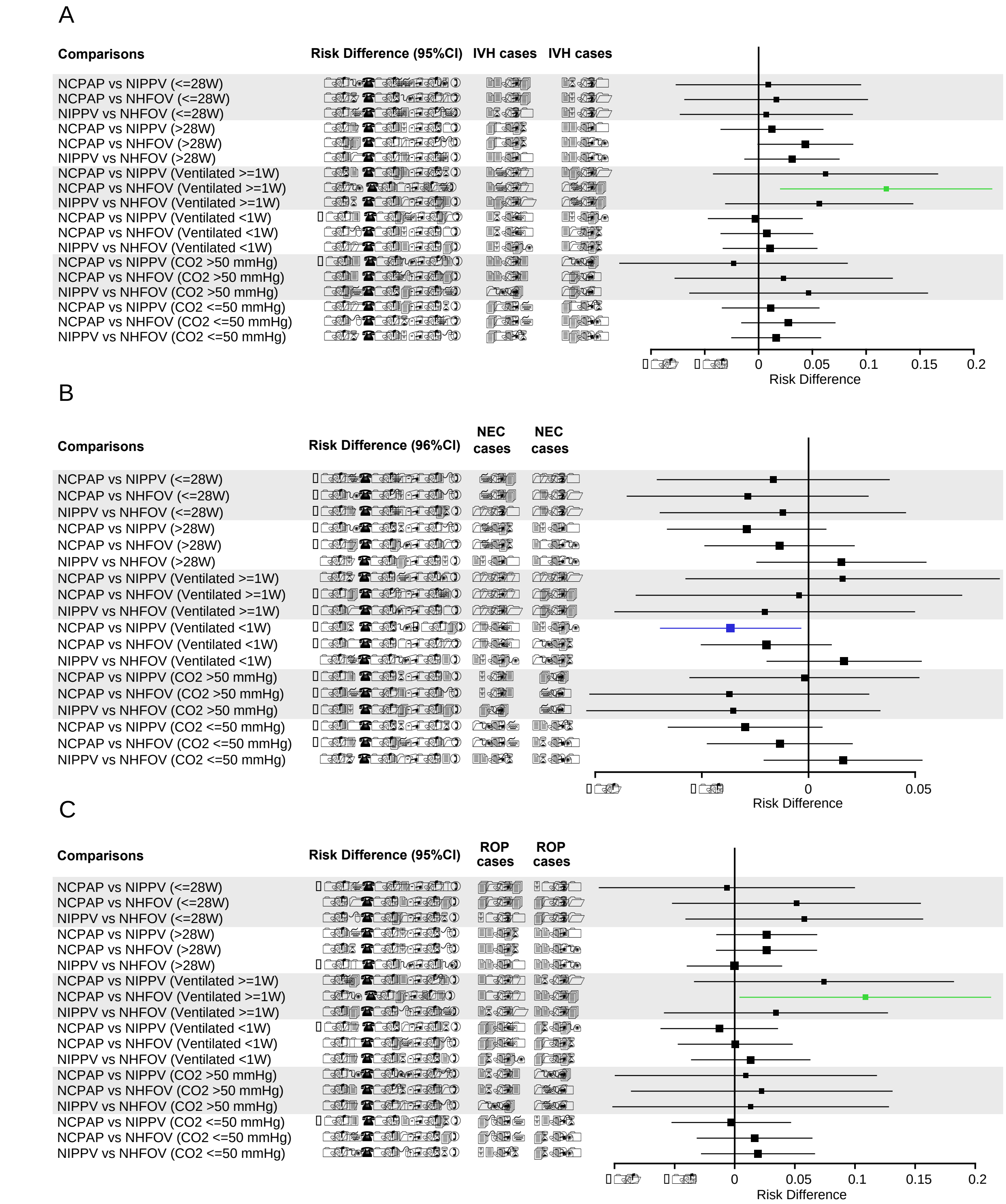

© 2023 Zhu X et al. *JAMA Network Open.*

eFigure 7. Secondary Outcomes: Duration of Oxygen Supplementation (A) and of Study Intervention (B). Subgroups of interest are highlighted with a grey background, alternate subgroups have no background. Both outcomes are measured in days. Data are shown as mean difference and 95% confidence interval. Data are illustrated as Forrest plots per each subgroup. Squares and lines indicate the mean differences and their 95% confidence interval, respectively. Square size is proportional to the subgroup numerosity. Green and red lines indicate comparisons significantly in favor of NHFOV and NCPAP, respectively. **Abbreviations**: CI: confidence interval; NCPAP: nasal continuous positive airway pressure; NHFOV: non-invasive high-frequency oscillation ventilation; NIPPV: noninvasive positive pressure ventilation.

A

Comparisons	Mean Difference (96%CI)
NCPAP vs NIPPV (<=28W)	-1.000 (-6.373, 4.373)
NCPAP vs NHFOV (<=28W)	3.000 (-2.137, 8.137)
NIPPV vs NHFOV (<=28W)	4.000 (-1.475, 9.475)
NCPAP vs NIPPV (>28W)	0.000 (-2.291, 2.291)
NCPAP vs NHFOV (>28W)	2.000 (-0.130, 4.130)
NIPPV vs NHFOV (>28W)	2.000 (-0.331, 4.331)
NCPAP vs NIPPV (Ventilated >1W)	0.000 (-6.187, 6.187)
NCPAP vs NHFOV (Ventilated >1W)	4.000 (-1.436, 9.436)
NIPPV vs NHFOV (Ventilated >1W)	6.000 (0.167, 11.833)
NCPAP vs NIPPV (Ventilated <1W)	-1.000 (-3.721, 1.721)
NCPAP vs NHFOV (Ventilated <1W)	1.000 (-1.647, 3.647)
NIPPV vs NHFOV (Ventilated <1W)	2.000 (-0.900, 4.900)
NCPAP vs NIPPV (CO2 >50 mmHg)	-2.000 (-8.581, 4.581)
NCPAP vs NHFOV (CO2 >50 mmHg)	0.000 (-5.486, 5.486)
NIPPV vs NHFOV (CO2 >50 mmHg)	2.000 (-5.132, 9.132)
NCPAP vs NIPPV (CO2 <=50 mmHg)	-2.000 (-4.806, 0.806)
NCPAP vs NHFOV (CO2 <=50 mmHg)	0.000 (-2.725, 2.725)
NIPPV vs NHFOV (CO2 <=50 mmHg)	2.000 (-0.886, 4.886)

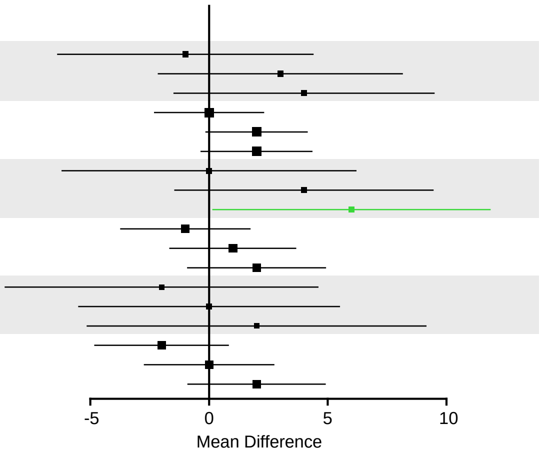

B

Comparisons	Mean Difference (95%CI)
NCPAP vs NIPPV (<=28W)	-2.000 (-5.902, 1.902)
NCPAP vs NHFOV (<=28W)	0.000 (-3.538, 3.538)
NIPPV vs NHFOV (<=28W)	2.000 (-2.062, 6.062)
NCPAP vs NIPPV (>28W)	-1.000 (-2.449, 0.449)
NCPAP vs NHFOV (>28W)	2.000 (0.708, 3.292)
NIPPV vs NHFOV (>28W)	3.000 (1.596, 4.404)
NCPAP vs NIPPV (Ventilated >1W)	0.000 (-4.041, 4.041)
NCPAP vs NHFOV (Ventilated >1W)	2.000 (-1.671, 5.671)
NIPPV vs NHFOV (Ventilated >1W)	2.000 (-1.754, 5.754)
NCPAP vs NIPPV (Ventilated <1W)	-2.000 (-3.932, -0.068)
NCPAP vs NHFOV (Ventilated <1W)	0.000 (-1.770, 1.770)
NIPPV vs NHFOV (Ventilated <1W)	2.000 (-0.086, 4.086)
NCPAP vs NIPPV (CO2 >50 mmHg)	-2.000 (-6.486, 2.486)
NCPAP vs NHFOV (CO2 >50 mmHg)	0.000 (-3.485, 3.485)
NIPPV vs NHFOV (CO2 >50 mmHg)	2.000 (-2.971, 6.971)
NCPAP vs NIPPV (CO2 <=50 mmHg)	-2.000 (-3.941, -0.059)
NCPAP vs NHFOV (CO2 <=50 mmHg)	1.000 (-0.866, 2.866)
NIPPV vs NHFOV (CO2 <=50 mmHg)	3.000 (1.099, 4.901)

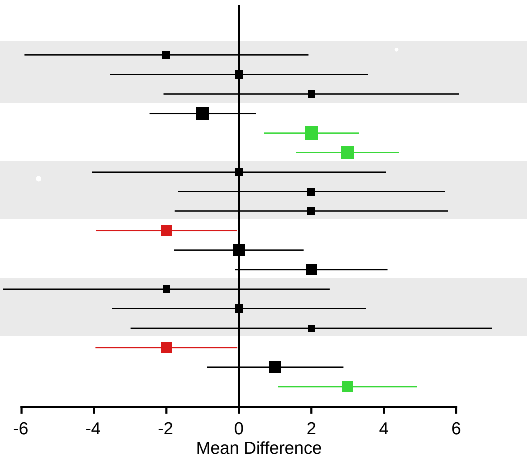

© 2023 Zhu X et al. *JAMA Network Open.*
