# Supplementary material for: Effectiveness of Nasal Continuous Positive Airway Pressure vs Nasal Intermittent Positive Pressure Ventilation vs Noninvasive High-Frequency Oscillatory Ventilation as Support After Extubation of Neonates Born Extremely Preterm or With More Severe Respiratory Failure: A Secondary Analysis of a Randomized Clinical Trial
Source: JAMA Netw Open. 2023 Jul 3;6(7):e2321644. doi: 10.1001/jamanetworkopen.2023.21644 (PMC10318479; doi:10.1001/jamanetworkopen.2023.21644)
Supplement: Supplement 3. — Nonauthor Collaborators [file jamanetwopen-e2321644-s003.pdf]

*Indicates required information. Only first name, last name, and suffix will appear in PubMed.

*Group Name(s): Nasal Oscillation Post-Extubation (NASONE) Study Group							
***First Name and Middle Initial(s)**	***Last Name**	***Suffix (eg, Jr, III)**	Academic Degrees	Institution	Location (city, state/province, country)	Role or Contribution, eg, chair, principal investigator	Group (if more than 1 Group listed in the byline) and/or Subgroup (eg, Steering Committee)
Xiaoyun	Zhong		MD	Department of neonatology, Chongqing Women and Children Health Hospital	Chongqing,China	investigator	
Sijie	Song		PhD, MD	Department of neonatology, Chongqing Women and Children Health Hospital	Chongqing,China	investigator	
Lan	Zhang		MD	Anhui province affiliated Hospital of Anhui medical university	Hefei/Anhui, China	investigator	
Li	Li		MD	Children's Hospital of The Capital Institute of Pediatrics	Beijing, China	investigator	
Huiqiang	Liu		MD	Third Hospital, Peking University.	Beijing, China	investigator	
Xiaomei	Tong		PhD, MD	Third Hospital, Peking University.	Beijing, China	investigator	
Xiaojing	Xu		MD	the first affiliated hospital of Tsinghua University	Beijing, China	investigator	
LiFeng	Cui		PhD, MD	the first affiliated hospital of Tsinghua University	Beijing, China	investigator	
Ming	Yi		MD	Women and children's hospital, branch of Chongqing sanxia central hospital	Chongqing,China	investigator	
Zhoujie	Peng		MD	Women and children's hospital, branch of Chongqing sanxia central hospital	Chongqing,China	investigator	
Jie	Li		MD	The First Affiliated Hospital of Chongqing Medical University	Chongqing,China	investigator	
Dongmei	Chen		MD	Department of Neonatal Intensive Care Unit, Quanzhou Children's Hospital	Quanzhou/Fujian,China	investigator	
Weifeng	Zhang		MD	Department of Neonatal Intensive Care Unit, Quanzhou Children's Hospital	Quanzhou/Fujian,China	investigator	

Supplemental Online Content: Nonauthor Collaborators

*Indicates required information. Only first name, last name, and suffix will appear in PubMed.

***First Name and Middle Initial(s)**	***Last Name**	***Suffix (eg, Jr, III)**	Academic Degrees	Institution	Location (city, state/province, country)	Role or Contribution, eg, chair, principal investigator	Group (if more than 1 Group listed in the byline) and/or Subgroup (eg, Steering Committee)
Xinzhu	Lin		PhD, MD	Department of Neonatology, Maternal and Child Health Care Hospital of Xiamen	Xiamen/Fujian, China	investigator	
Bin	Wang		PhD,MD	Neonatology and Pediatrics Department, Zhujiang Hospital, Southern Medical University	Guangzhou/Guangdong, China	investigator	
Weimin	Huang		MD	Department of Neonatology, Nanfang Hospital, Southern Medical University	Guangzhou/Guangdong, China	investigator	
Guangliang	Bi		MD	Department of Neonatology, Nanfang Hospital, Southern Medical University	Guangzhou/Guangdong, China	investigator	
Shaoru	He		MD	Department of Neonatology, Guangdong Academy of Medical Science/Guangdong General Hospital	Guangzhou/Guangdong, China	investigator	
Yumei	Liu		MD	Department of Neonatology, Guangdong Academy of Medical Science/Guangdong General Hospital	Guangzhou/Guangdong, China	investigator	
Jie	Yang		MD	Department of Neonatology, Guangdong Women and Children Hospital	Guangzhou/Guangdong, China	investigator	
Weiwei	Gao		MD	Department of Neonatology, Guangdong Women and Children Hospital	Guangzhou/Guangdong, China	investigator	
Wuhua	Liang		MD	Department of Neonatology, Women and Children's Health Hospital of Yulin	Yulin/Guangxi Zhuang Autonomous Regioni, China	investigator	

*Indicates required information. Only first name, last name, and suffix will appear in PubMed.

***First Name and Middle Initial(s)**	***Last Name**	***Suffix (eg, Jr, III)**	Academic Degrees	Institution	Location (city, state/province, country)	Role or Contribution, eg, chair, principal investigator	Group (if more than 1 Group listed in the byline) and/or Subgroup (eg, Steering Committee)
Yaoxun	Wu		MD	Department of Neonatology, Women and Children's Health Hospital of Yulin	Yulin/Guangxi Zhuang Autonomous Regioni, China	investigator	
Xinnian	Pan		PhD, MD	Department of Neonatology, Maternal and Children health care hospital of Guangxi Zhuang Autonomous Region	Nanning/Guangxi Zhuang Autonomous Regioni, China	investigator	
Qiufen	Wei		PhD, MD	Department of Neonatology, Maternal and Children health care hospital of Guangxi Zhuang Autonomous Region	Nanning/Guangxi Zhuang Autonomous Regioni, China	investigator	
Yujun	Chen		PhD, MD	Department of Pediatrics, the Second Affiliated Hospital of Guangxi Medical University	Nanning/Guangxi Zhuang Autonomous Regioni, China	investigator	
Bingmei	Wei		MD	Department of Pediatrics, the Second Affiliated Hospital of Guangxi Medical University	Nanning/Guangxi Zhuang Autonomous Regioni, China	investigator	
Ling	Liu		PhD, MD	Department of Neonatology, Guiyang Maternal and Child Health Care Hospital	Guiyang/Guizhou,China	investigator	
Xinghui	Zheng		MD	Department of Neonatology, The first people's Hospital of Zunyi	Zunyi/Guizhou, China	investigator	
Ding	Xu		MD	Department of Neonatology, the Second Hospital of Lanzhou University	Lanzhou/Gansu, China	investigator	
Fan	Wang		MD	Department of Neonatology, the Second Hospital of Lanzhou University	Lanzhou/Gansu, China	investigator	
Bin	Yi		MD	Department of neonatology, GanSu provencial Maternity and Child Care hospital	Lanzhou/Gansu, China	investigator	

*Indicates required information. Only first name, last name, and suffix will appear in PubMed.

***First Name and Middle Initial(s)**	***Last Name**	***Suffix (eg, Jr, III)**	Academic Degrees	Institution	Location (city, state/province, country)	Role or Contribution, eg, chair, principal investigator	Group (if more than 1 Group listed in the byline) and/or Subgroup (eg, Steering Committee)
Jingyun	Shi		MD	Department of neonatology, GanSu provencial Maternity and Child Care hospital	Lanzhou/Gansu, China	investigator	
Yuning	Li		MD	Department of Pediatrics, The First Hospital of Lanzou University	Lanzhou/Gansu, China	investigator	
Li	Jiang		MD	Department of Pediatrics, The First Hospital of Lanzou University	Lanzhou/Gansu, China	investigator	
Chunming	Jiang		MD	The First Affiliated Hospital of Harbin Medical University	Harbin/Heilongjiang, China	investigator	
Chenghe	Tang		MD	The First Affiliated Hospital of Xinxiang Medical University	Xinxiang/Henan, China	investigator	
Hong	Xiong		MD	Neonatal Intensive Care Unit and Department of Neonatology, Zhengzhou Children's Hospital, the Children's Hospital of Zhengzhou Universit	Zhengzhou/Henan, China	investigator	
Huiqing	Sun		MD	Neonatal Intensive Care Unit and Department of Neonatology, Zhengzhou Children's Hospital, the Children's Hospital of Zhengzhou Universit	Zhengzhou/Henan, China	investigator	
Wenqing	Kang		MD	Neonatal Intensive Care Unit and Department of Neonatology, Zhengzhou Children's Hospital, the Children's Hospital of Zhengzhou Universit	Zhengzhou/Henan, China	investigator	
Dapeng	Liu		MD	Neonatal Intensive Care Unit and Department of Neonatology, Zhengzhou Children's Hospital, the Children's Hospital of Zhengzhou Universit	Zhengzhou/Henan, China	investigator	

Supplemental Online Content: Nonauthor Collaborators

*Indicates required information. Only first name, last name, and suffix will appear in PubMed.

*First Name and Middle Initial(s)	*Last Name	*Suffix (eg, Jr, III)	Academic Degrees	Institution	Location (city, state/province, country)	Role or Contribution, eg, chair, principal investigator	Group (if more than 1 Group listed in the byline) and/or Subgroup (eg, Steering Committee)
Falin	Xu		PhD, MD	Department of Neonatology,The Third affiliated Hospital of Zhengzhou University	Zhengzhou/Henan, China	investigator	
Kaihui	Xing		MD	Department of Neonatal Intensive Care Unit, the Maternal and child health hospital of Hainan province	Haikou/Hainan, China	investigator	
Ning	Yang		MD	Department of Neonatal Intensive Care Unit, the Maternal and child health hospital of Hainan province	Haikou/Hainan, China	investigator	
Fang	Liu		MD	Department of Neonatal Intensive Care Unit, Bethune International Peace Hospital	Shijiazhuang/Hebei, China	investigator	
Shaoguang	Lv		MD	Department of Neonatal Intensive Care Unit, Bethune International Peace Hospital	Shijiazhuang/Hebei, China	investigator	
Hanchu	Liu		MD	Department of Neonatology, Wuhan Children’s Hospital, Tongji Medical College, Huazhong University of Science & Technology	Wuhan/Hubei, China	investigator	
Wenchao	Yuan		MD	Department of Neonatology, Wuhan Children’s Hospital, Tongji Medical College, Huazhong University of Science & Technology	Wuhan/Hubei, China	investigator	
Rui	Cheng		MD	Department of Neonatology, Children's Hospital of Nanjing Medical University	Nanjing/Jiangsu. China	investigator	

*Indicates required information. Only first name, last name, and suffix will appear in PubMed.

***First Name and Middle Initial(s)**	***Last Name**	***Suffix (eg, Jr, III)**	Academic Degrees	Institution	Location (city, state/province, country)	Role or Contribution, eg, chair, principal investigator	Group (if more than 1 Group listed in the byline) and/or Subgroup (eg, Steering Committee)
Xian	Shen		MD	Department of Neonatology, Children's Hospital of Nanjing Medical University	Nanjing/Jiangsu. China	investigator	
Hui	Wu		MD	Department of Neonatology, the First Hospital of Jilin University	Changchun/Jilin, China	investigator	
Laishuan	Wang		PhD, MD	Department of neonatology, Children's hospital of Fudan University	Shanghai, China	investigator	
Zhenying	Yang		MD	Department of Neonatology, Maternal and Children's Healthcare Hospital of Taian	Taian/Shandong, China	investigator	
Xiao	Zhang		MD	Department of Neonatology, Maternal and Children's Healthcare Hospital of Taian	Taian/Shandong, China	investigator	
Jiang	Xue		MD	Department of neonatal intensive care unit, the second hospital of Shandong University	Jinan/Shandong, China	investigator	
Zhankui	Li		MD	Department of Neonatology, North-West Women and Children's Hospital	Xi'an/Shanxi, China	investigator	
Rong	Ju		MD	Department of Neonatal Intensive Care Unit, the Women and Children's \Center Hospital of Chengdu	Chengdu/Sichuan, China	investigator	
Jin	Wang		MD	Department of Neonatal Intensive Care Unit, the Women and Children's \Center Hospital of Chengdu	Chengdu/Sichuan, China	investigator	
Wenbin	Dong		MD	Department of Newborn Medicine, the Affiliated Hospital of Southwest Medical University	Luzhou/Sichuan, China	investigator	

*Indicates required information. Only first name, last name, and suffix will appear in PubMed.

*First Name and Middle Initial(s)	*Last Name	*Suffix (eg, Jr, III)	Academic Degrees	Institution	Location (city, state/province, country)	Role or Contribution, eg, chair, principal investigator	Group (if more than 1 Group listed in the byline) and/or Subgroup (eg, Steering Committee)
Xiaoxiu	Ye		MD	Department of Newborn Medicine,Hospital Affiliated to Southwest Medical University	Luzhou/Sichuan, China	investigator	
Benqing	Wu		MD	Department of Neonatology, Shenzhen People's hospital/the Second Medical College of Jinan University	Shenzhen/Guangdong, China	investigator	
Jun	Zheng		MD	Department of Neonatology, Tianjin Central Hospital of Obstetrics and Gynecology	Tianjin, China	investigator	
Xiuying	Tian		MD	Department of Neonatology, Tianjin Central Hospital of Obstetrics and Gynecology	Tianjin, China	investigator	
Mingxia	Li		MD	Department of Neonatology, the first affiliated hospital of Xinjiang Medical Universit	Urumqi/Xinjiang Uygur Autonomous Region, China	investigator	
Yanping	Zhu		MD	Department of Neonatology, the first affiliated hospital of Xinjiang Medical Universit	Urumqi/Xinjiang Uygur Autonomous Region, China	investigator	
Nuerya	Rejiafu		MD	Department of Neonatology, People's Hospital of Xinjiang Uygur Autonomous Region	Urumqi/Xinjiang Uygur Autonomous Region, China	investigator	
Long	Li		MD	Department of Neonatology, People's Hospital of Xinjiang Uygur Autonomous Region	Urumqi/Xinjiang Uygur Autonomous Region, China	investigator	
Yangfang	Li		MD	Department of neonatology, children's hospital of Kunming medical university	Kunming/Yunnan, China	investigator	
Canlin	He		MD	Department of neonatology, children's hospital of Kunming medical university	Kunming/Yunnan, China	investigator	

Supplemental Online Content: Nonauthor Collaborators

*Indicates required information. Only first name, last name, and suffix will appear in PubMed.

*First Name and Middle Initial(s)	*Last Name	*Suffix (eg, Jr, III)	Academic Degrees	Institution	Location (city, state/province, country)	Role or Contribution, eg, chair, principal investigator	Group (if more than 1 Group listed in the byline) and/or Subgroup (eg, Steering Committee)
Li	Li		MD	Pediatric Department, the first people's hospital of Yunnan province	Kunming/Yunnan, China	investigator	
Hongying	Mi		MD	Pediatric Department, the first people's hospital of Yunnan province	Kunming/Yunnan, China	investigator	
Kun	Liang		PhD, MD	Department of Pediatrics, the first affiliated hospital of Kunming Medical University	Kunming/Yunnan, China	investigator	
Hong	Cao		MD	Department of Neonatal Intensive Care Unit, Yan'an Hospital Affiliated to Kunming Medical University	Kunming/Yunnan, China	investigator	
Linlin	Xia		MD	Department of Neonatal Intensive Care Unit, Yan'an Hospital Affiliated to Kunming Medical University	Kunming/Yunnan, China	investigator	
Chuanfeng	Li		MD	Department of Neonatology, Women and Children's Health Hospital of Qujing	Qujing/Yunnan, China	investigator	
Zhaoqing	Yin		MD	Department of neonatology,The people's hospital of Dehong autonomous prefecture	Dehong autonomous prefecture/Yunan, China	investigator	
Le	Su		MD	Department of neonatology,The people's hospital of Dehong autonomous prefecture	Dehong autonomous prefecture/Yunan, China	investigator	
Yanxiang	Chen		MD	Department of Neonatology, Yinchuan No.1 people's Hospital Affiliated of Ningxia Medical University	Yinchuan/Ningxia Hui Autonomous Region, China	investigator	

Supplemental Online Content: Nonauthor Collaborators

*Indicates required information. Only first name, last name, and suffix will appear in PubMed.

***First Name and Middle Initial(s)**	***Last Name**	***Suffix (eg, Jr, III)**	Academic Degrees	Institution	Location (city, state/province, country)	Role or Contribution, eg, chair, principal investigator	Group (if more than 1 Group listed in the byline) and/or Subgroup (eg, Steering Committee)
Liping	Shi		PhD, MD	Department of Neonatal Intensive Care Unit, The Children's Hospital of Zhejiang University School of Medicine	Hangzhou/Zhejiang, China	investigator	
Chenhong	Wang		MD	Department of Neonatal Intensive Care Unit, The Children's Hospital of Zhejiang University School of Medicine	Hangzhou/Zhejiang, China	investigator	
Jiajun	Zhu		PhD, MD	Department of Neonatology, Women's Hospital, School of Medicine, Zhejiang Universit	Hangzhou/Zhejiang, China	investigator	
Xuefeng	Zhang		MD	Department of Neonatology, 302 Military hospital of china, People Liberation Army of China	Beijing, China	investigator	
Xirong	Gao		MD	Department of Neonatology, Hunan Children's hospital	Changsha/Hunan, China	investigator	
Bo	Lv		MD	Department of Neonatology, Hunan Children's hospital.	Changsha/Hunan, China	investigator	
Chongde	Liu		MD	Department of Neonatology,Women and Children's Hospital of Qinghai	Xining/Qinghai, China	investigator	
Xiaorong	Wang		MD	Department of Neonatology,Women and Children's Hospital of Qinghai	Xining/Qinghai, China	investigator	
Liping	Chen		MD	Department of Neonatology, Jiangxi Provincial Children's Hospital	Nanchang/Jiangxi, China	investigator	
Lin	Li		MD	Department of Neonatology, Jiangxi Provincial Children's Hospital	Nanchang/Jiangxi, China	investigator	

*Indicates required information. Only first name, last name, and suffix will appear in PubMed.

*First Name and Middle Initial(s)	*Last Name	*Suffix (eg, Jr, III)	Academic Degrees	Institution	Location (city, state/province, country)	Role or Contribution, eg, chair, principal investigator	Group (if more than 1 Group listed in the byline) and/or Subgroup (eg, Steering Committee)
Chunli	Zhang		MD	Department of Neonatology,Inner Mongolia People's Hospital	Hohhot/Inner Mongolia, China.	investigator	
Jia	Chen		MD	Department of Neonatology, Mianyang central hospital	Mianyang/Sichuan, China	investigator	
Qiyu	Li		MD	Department of Pediatrics, 202 Military hospital of People's Liberation Army of China	Shenyang/Liaoning, China	investigator	
Qin	Lv		MD	Infant Intensive Care Unit, Ningbo women and children hospital	Ningbo/Zhejiang, China	investigator	
Yanhong	Li		MD	Infant Intensive Care Unit, Ningbo women and children hospital	Ningbo/Zhejiang, China	investigator	
Yong	Ji		MD	Children’s hospital of Shanxi	Taiyuan/Shanxi, China	investigator	
Yanjiang	Chen		MD	Children’s hospital of Shanxi	Taiyuan/Shanxi, China	investigator	
Jianhua	Sun		PhD, MD	Department of Neonatology, Shanghai Children’s Medical Center	Shanghai, China	investigator	
Jun	Bu		MD	Department of Neonatology, Shanghai Children’s Medical Center	Shanghai, China	investigator	
Danni	Zhong		PhD, MD	Department of Pediatrics,The First Affiliated Hospital of Guangxi Medical University	Nanning/Guangxi Zhuang Autonomous Regioni, China	investigator	
Zongyan	Cao		MD	Department of Pediatrics,The First Affiliated Hospital of Guangxi Medical University	Nanning/Guangxi Zhuang Autonomous Regioni, China	investigator	
Shuping	Han		MD	Department of Pediatrics, Obstetrics and Gynecology Hospital of Affiliated to Nanjing Medical University	Nanjing/Jiangsu. China	investigator	
Xiaohui	Chen		MD	Department of Pediatrics, Obstetrics and Gynecology Hospital of Affiliated to Nanjing Medical University	Nanjing/Jiangsu. China	investigator	

Supplemental Online Content: Nonauthor Collaborators

*Indicates required information. Only first name, last name, and suffix will appear in PubMed.

*First Name and Middle Initial(s)	*Last Name	*Suffix (eg, Jr, III)	Academic Degrees	Institution	Location (city, state/province, country)	Role or Contribution, eg, chair, principal investigator	Group (if more than 1 Group listed in the byline) and/or Subgroup (eg, Steering Committee)
Caiyun	Gao		MD	Pediatric Department, Qinhuangdao maternal and child health care hospital	Qinhuangdao/Hebei, China	investigator	
Hongbin	Zhu		MD	Pediatric Department, Qinhuangdao maternal and child health care hospital	Qinhuangdao/Hebei, China	investigator	
Zhenguang	Li		MD	Xuzhou Children Hospital	Xuzhou/Jiangsu, China	investigator	
Hongwei	Wu		MD	Xuzhou Children Hospital.	Xuzhou/Jiangsu, China	investigator	
Xiuyong	Cheng		PhD, MD	The first affiliated hospital of Zhengzhou university	Zhengzhou/Henan, China	investigator	
Juhua	Li		MD	Department of Pediatrics, Xianyang children's Hospital	Xianyang/Shanxi, China	investigator	
Long	Chen		MD	Department of Pediatrics, Daping hospital of Third Military Medical University	Chongqing, China	investigator	
Huanhuan	Li		MD	Department of Neonatology JiulongpoPeople's Hospital	Chongqing, China	investigator	
