# Supplementary material for: Effectiveness of Nasal Continuous Positive Airway Pressure vs Nasal Intermittent Positive Pressure Ventilation vs Noninvasive High-Frequency Oscillatory Ventilation as Support After Extubation of Neonates Born Extremely Preterm or With More Severe Respiratory Failure: A Secondary Analysis of a Randomized Clinical Trial
Source: JAMA Netw Open. 2023 Jul 3;6(7):e2321644. doi: 10.1001/jamanetworkopen.2023.21644 (PMC10318479; doi:10.1001/jamanetworkopen.2023.21644)
Supplement: Supplement 4. — Data Sharing Statement [file jamanetwopen-e2321644-s004.pdf]

# Data Sharing Statement

Zhu. Effectiveness of Nasal Continuous Positive Airway Pressure vs Nasal Intermittent Positive Pressure Ventilation vs Noninvasive High-Frequency Oscillatory Ventilation as Support After Extubation of Neonates Born Extremely Preterm or With More Severe Respiratory Failure. *JAMA Netw Open*. Published July 03, 2023. doi:10.1001/jamanetworkopen.2023.21644

**Data**

**Data available:** The raw data are available upon reasonable request.
